# Dietary exposure to potentially harmful elements in edible plants in Poland and the health risk dynamics related to their geochemical differentiation

**DOI:** 10.1038/s41598-023-35647-x

**Published:** 2023-05-25

**Authors:** Agata Wódkowska, Agnieszka Gruszecka-Kosowska

**Affiliations:** Department of Environmental Protection, Faculty of Geology, Geophysics, and Environmental Protection, AGH University of Science and Technology, Al. Mickiewicza 30, 30-059 Kraków, Poland

**Keywords:** Element cycles, Environmental chemistry, Environmental impact

## Abstract

Differences in the health risk values calculated for consumers of potentially harmful elements (PHEs) present in edible plants were investigated. Based on a comprehensive literature search, the highest PHE contents in plants were identified in the southern and western regions of Poland, that also revealed the highest geochemical enrichment with Zn, Pb, Cu, As, Cd, and Tl. The highest unacceptable non-carcinogenic risk (HQ) values for mean PHE contents in Poland were found for Pb: toddlers (2.80), pre-schoolers (1.80), and school-aged children (1.45) and for Cd for toddlers (1.42). The highest unacceptable carcinogenic risk (CR) values for mean As content was observed for adults (5.9 × 10^–5^). The highest non-carcinogenic risk values for consumers were reported in Silesia, Lower Silesia, Lublin, Lesser Poland, and Opole Provinces, indicating the impact of geochemical variability on risk values.

## Introduction

Edible land plants have always been an important part of the human diet, providing energy and nutrients for a balanced life^1^. The World Health Organization (WHO) recommends vegetables, fruits, legumes, nuts, and whole grains to be the main meal components, and the first two should be eaten at the amount of 400 g daily at least^2^. According to the Healthy Eating Plate^3^ guidelines each meal should comprise 30% vegetables, 25% whole grains, 25% healthy proteins, and 20% of fruits. National Institute of Public Health (PZH) in Poland from 2020 recommends that vegetables and fruits should constitute half and cereal products one fourth of each daily meal^4^.

Vegetables and fruits are an excellent source of minerals, necessary fatty acids, and fibre, but also a unique source of vitamins (C, E, K, and folates)^5^. At the same time, their calorific value, saturated fat and sodium contents are low, and they do not contain cholesterol^6^. The energy value of vegetables ranges from 8.4 to 74 kcal per 100 g, with an average value of only 26 kcal per 100 g^7^. This is particularly important considering that being overweight and obese are serious public health problems worldwide^8^. The nutrients provided by grains include carbohydrate/starch (energy), protein, fibre, and a wide variety of vitamins and minerals, including group B vitamins (folates, thiamine, riboflavin, niacin), vitamin E, iron, zinc, magnesium, and phosphorus^9,10^. The high fibre content in whole grain cereals also supports the functioning of the digestive system and can prevent constipation^11–13^. The consumption of fruits and vegetables, as well as grains is strongly associated with a reduced risk of cardiovascular diseases, cancer, diabetes, Alzheimer’s disease, cataracts, diverticular disease, and age-related impairment of body functions^14–18^.

Potentially Harmful Elements (PHEs) are widely present and dispersed in the environment. Their accumulation in plants is particularly important because nutritional substances might translocate from plants in the food chain and finally, they can accumulate in humans^19^. Due to the high nutritional importance of edible plants and their key role in the diet, the increased content of PHEs might pose a significant health risk for their consumers. Most of the PHEs entering the human body through the consumption pathway originate from the plant products grown in soil, which as the results of the geogenic or anthropogenic factors, may pose a threat of their migration to edible plants due to increased concentration or mobility^19^. Most research investigated the functioning of the elements in living organisms, but growing evidence suggests that the interactions between them are more complex than originally thought^20^. This is due to the possible synergistic and/or antagonistic interactions between them, but also due to the complex metabolic reactions occurring in the living organisms and the interactions with the human microbiome^21–23^. As health increasingly becomes more important for the society, food research related more closely with preventive medicine is gaining popularity^19,24–33^.

The occurrence of PHEs in the environment has continuously increased over the past decades^34^. The widespread interest in PHEs has only grown as a significant scientific topic in the last 50 years, when it became clear that some elements are essential to human health (e.g., Cu, Fe, and Zn), while others are toxic (e.g., As, Hg, and Pb) and might trigger adverse health effects^35^. The spectrum of toxic effects caused by PHEs is very broad^36,37^. Exposure to Cd can cause flu-like symptoms and can damage the lungs^38^, can have effects such as lung cancer, prostate proliferative changes, bone fractures, kidney dysfunction, and hypertension^38,39^. Rate and occurrence of neurotoxic effects of Hg depend on exposure factors like geochemical form of Hg, health conditions, and exposure characteristic^40,41^. The most severe effects of Hg exposure are neurological damage (mercurialism), asthenic-vegetative syndrome, and Minamata disease^42–44^. Due to its metabolism process As might damage each human body organ^45,46^. Exposure to Pb can cause plumbism, anaemia, gastrointestinal colic, and central nervous system (CNS) disorders, with children showing signs of severe Pb toxicity at lower doses than adults^47–49^. Co constitute the central part of the vitamin B12 molecule^50,51^ however, its excessive doses in the body can cause cardiomyopathy, disrupt the thyroid gland, increase in functioning of the bone marrow, and inhibit the absorption of vitamin B12^20,51,52^. Cu is essential to support proper fetal growth, brain functioning, and wound healing^53^. Exposure to Cu mainly concern the gastrointestinal tract, liver, kidneys, hematopoietic, cardiovascular, and central nervous system^53–55^. Zn is an essential element for processes like gene expression, enzymatic reactions, immune function, protein and DNA synthesis, wound healing, growth, and development^56,57^. Excess Zn can lead to the deterioration of the immune system, reduction in HDL cholesterol, vomiting and nausea, loss of appetite, diarrhea, fever, and headaches^20,57^. Skin contact with Ni can cause adverse health effects, such as dermatitis, cardiovascular and kidney diseases, pulmonary fibrosis, lung and nose cancer, vomiting and nausea, cyanosis, gastrointestinal discomfort, weakness, oedema, and even death^20,58^. Moreover, As, Cd, Cr, Co, Ni, and Pb have been classified by the International Agency for Research on Cancer (IARC) to have a carcinogenic impact on humans^59^, causing among other skin, lung, bladder, kidney, and liver cancers^60^.

Poland’s southern and western regions were and are rich in coal and lignite deposits, respectively^61^. The southern and central regions were or still are heavily exploited for Cu in Lower Silesia Province, for Fe in Silesia, Łódź, the Holy Cross, Masovian, and Lower Silesia provinces and for Zn and Pb in Lesser Poland and Silesia provinces^62^. The exploitation and processing in these areas have caused heavy environmental pollution and landscape devastation^63–65^ with Zn, Pb, Cu, Fe being the main reason for exploitation and as well as with accompanying elements i.e., Tl, Sb, Cd, and As. Natural processes of rock and soil geological weathering occurring in these regions also contributed to the elevated levels of these elements in the environment. In their studies, Lis and Pasieczna^66^ indicated high differentiations in the element content in soils in various regions related to their geochemical variability. This is a particularly important consideration in estimating the level of pollution, by comparing of element concentrations in environmental compartments against the permissible level as defined in law or guidance documents. A simple averaging of the content of these elements from the regions with geochemically elevated concentrations (southern and western Poland) with those coming from regions of stable content (eastern and northern Poland) and using these results further in the risk calculations might lead to unjustifiable results and to false and perhaps, even dangerous conclusions.

Thus, in our research we investigated the variation of the total risk values calculated depending on whether average national or regional concentrations of PHEs were used. This led us to the hypothesis that as the PHE concentrations in edible plants should be higher in regions where the concentrations in soils are also elevated, the health risk for consumers in these regions should also be higher. Considering the above, the aim of this study was to analyse the diversity of PHE concentrations in edible plants in Poland and in its individual regions, based on a scientific literature review. Based on the obtained data, health risk to consumers for all of Poland, as well as for the individual regions were calculated and compared as the trend of healthy eating, including the consumption of fresh fruits and vegetables bought in the local market is gaining popularity. The detailed objectives of the study included: (1) characteristics of the PHE contents (As, Cd, Co, Cr, Cu, Hg, Ni, Pb, Sb, Tl, and Zn) in vegetables, fruits, and cereals cultivated in the various regions in Poland based on the results from scientific research available in the consulted databases, (2) determination of the consumption rates of edible plants in Poland in the investigated subpopulations based on the recommended consumption rates, (3) health risk assessment related with the PHE consumption regarding edible plants depending on the region of Poland and the investigated subpopulations.

## Materials and methods

### Literature research and selection criteria

**Figure 1:**
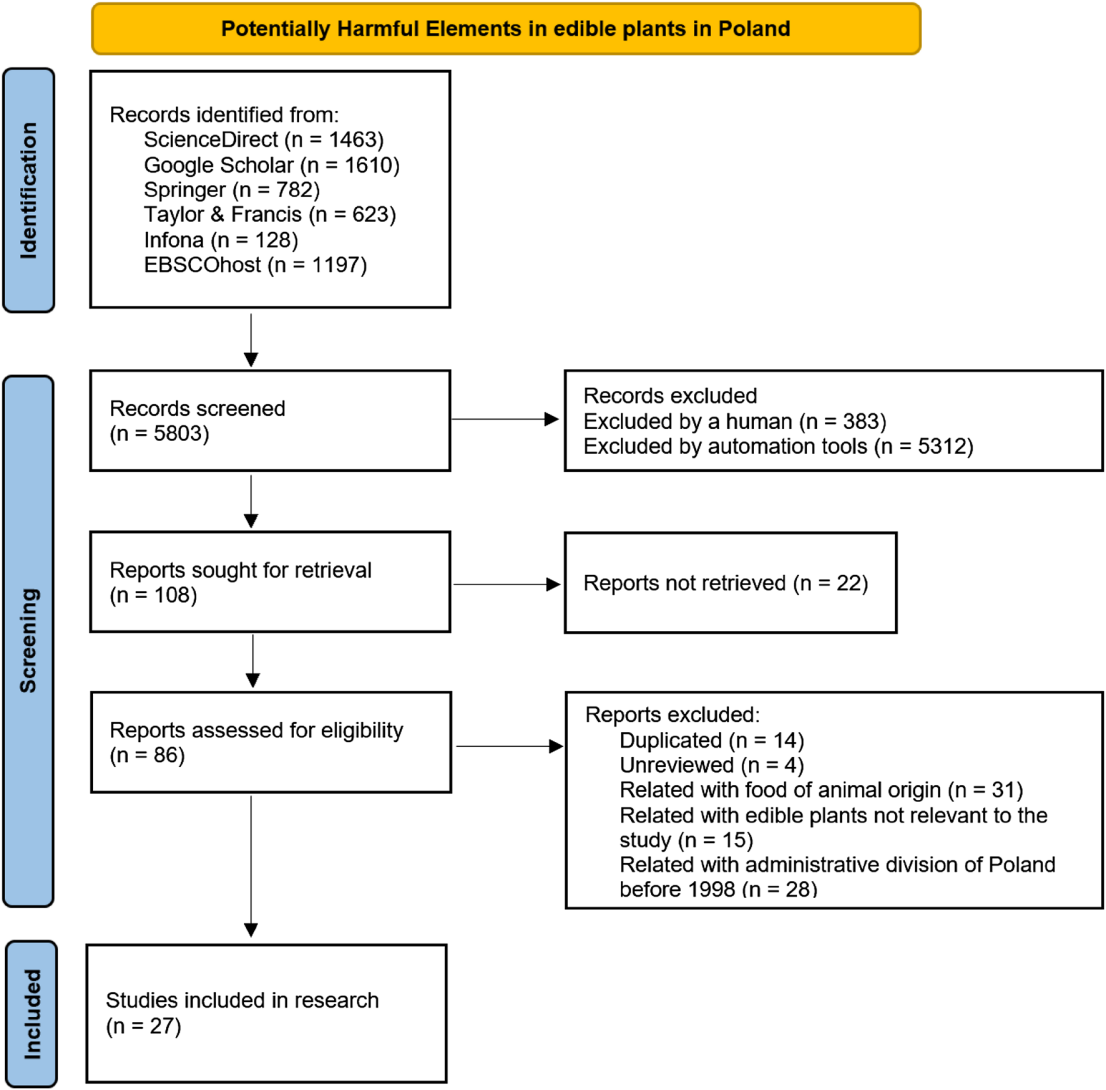
PRISMA diagram of the literature review on Potentially Harmful Elements concentrations in edible plants in Poland.

**Figure 2:**
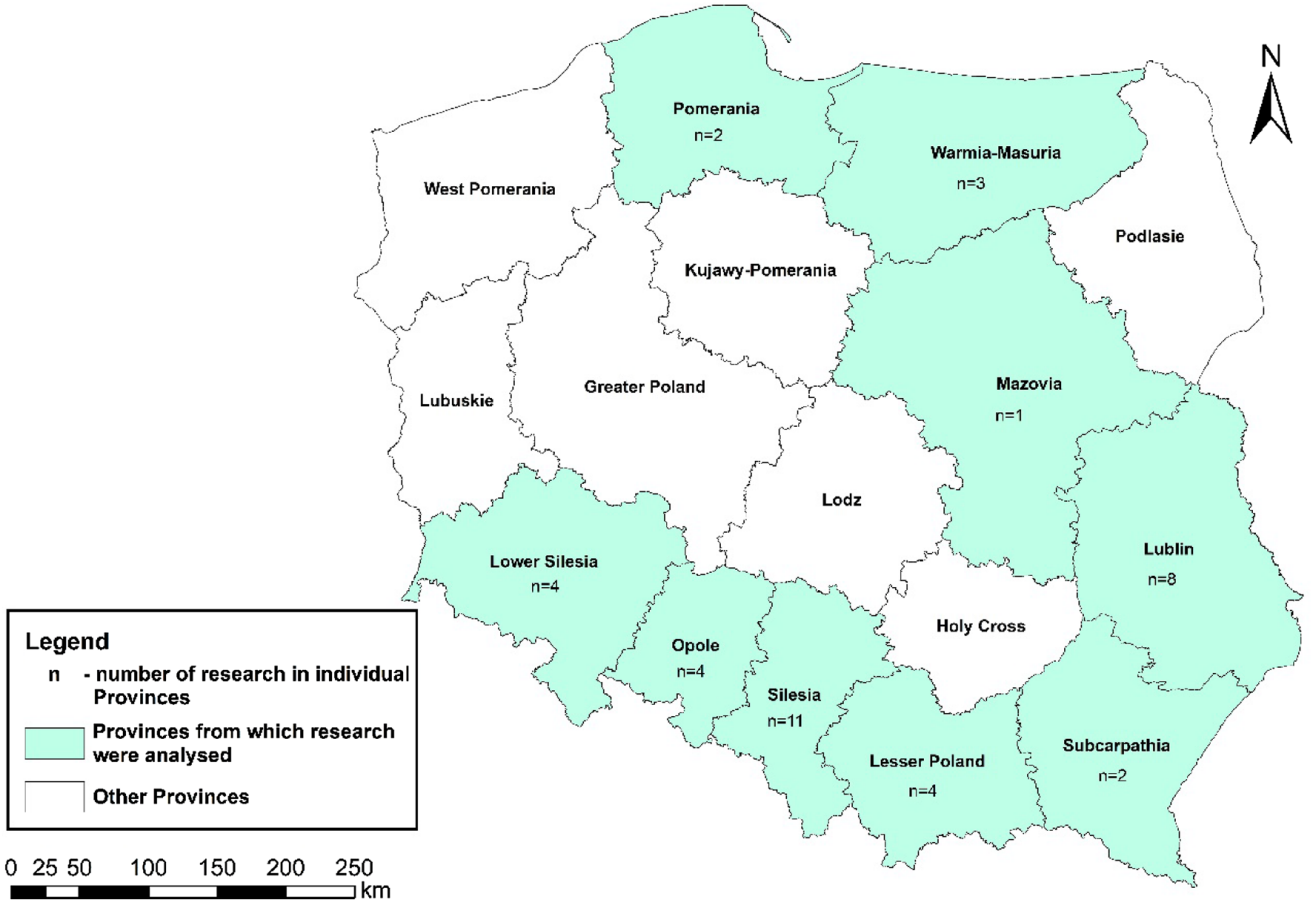
The localisation of research on PHE contents in edible plants in Poland (Esri ArcMap 10.8.0.12790; http://esri.com).

To collect concentrations of the investigated PHEs in edible plants cultivated in Poland, a comprehensive literature search in the timeframe from 1968 to 2021 was performed from February to March 2021 in the following databases: ScienceDirect, Google Scholar, Infona, EBSCOhost, Springer, and Taylor & Francis. Combinations of the following keywords were used for searching the results: potentially harmful elements, metals, heavy metals, edible plants, food, fruits, vegetables, cereals, Poland. In total 5803 records were found, all of which were checked for further utility according to the following aspects: (1) duplicate articles were removed; (2) unreviewed articles were not included; (3) research on the content of heavy metals in animal food products were not considered; (4) articles on research performed before 1998 in accordance with the previous geographical administrative division of Poland to provinces were not considered. All details of the literature review were presented on the PRISMA Flow Diagram^67^ in Fig. 1. During the process, 86 articles were selected, for which their abstracts were investigated regarding the relevance to our scientific topic. Based on the analysis of the following information (1) short bibliographic description (authors, title, year of publication); (2) investigated edible plants; (3) investigated PHEs; (4) methods of PHEs extraction and determination; and (5) localisation of the research area including the province name, in the main analysis, 27 articles were selected. As some research provided data for more than a single province, particular numbers of articles included in our study are presented in Fig. 2. Due to a low number of research in some of the provinces, data for Mazovia (n = 1) and Subcarpathia (n = 2) provinces were used only for calculating national contents of PHEs. Similarly, due to a low number of references in Pomerania (n = 2) and Warmia-Masuria (n = 3) in our research we joined these two provinces as Northern Poland (n = 5) prior further analysis. Regarding the types of edible plants that were investigated in research articles for our research, they were grouped as presented in Table 1. The number of references obtained for each PHE was as follows: As 6, Cd 23, Co 4, Cu 11, Hg 5, Ni 7, Pb 21, and Zn 11. As articles involved a various number of investigated plants and their locations, the different number of actual sources was reported.

**Table 1:** Grouping of the edible plants that were recognised in the literature review to be investigated in Poland.

Groups of edible plants analysed in Poland	
Vegetables	
Leaf	Arugula, chard, cabbage, celery, chives, kale, leek, lovage, parsley, spinach, lettuce, dill, Brussel sprouts
Fruit	Zucchini, pumpkin, cucumber, pepper, tomato, eggplant
Inflorescence	Broccoli, cauliflower
Legume	Green bean, broad bean, haricot, pea, green pea
Root	Potato, beet, carrot, celery, horseradish, radish, parsley, turnip
Fruits	
Berry	Black currant, blackberry, blueberry, gooseberry, grape, raspberry, red currant, strawberry, wild strawberry, cranberry, black lilac, chokeberry
Pome	Apple, pear, rosehip, hawthorn
Stone	Peach, cherry, gean, nectarine, plum, apricot
Cereals	Wheat, rye, barley, oat

### Extraction and determination methods of PHEs in edible plants defined in the literature research

The investigated methods should ideally be the same to compare the results received from multiple research studies. However, as there is no one mandatory methodology required/recommended for this type of analysis, before further using the obtained results, we compared the methods of PHE extraction to check if the results might be comparable with each other, in order to perform a reliable risk assessment further in the project. The list of the articles included to our research during the literature review together with the methods used in those studies is presented in Supplementary Table S1. As can be observed, extraction methods are commonly used for determining total or pseudo-total PHE concentrations in edible plants. Thus, the results from the analyses performed by these methods were further used in our studies. Moreover, the determination of the PHE contents in the extractants were performed using two of the most popular instrumental methods, Atomic Absorption Spectrometry (AAS) in the case of 19 publications and Inductively Coupled Plasma Mass Spectrometry in the case of 11 publications from the 27 analysed articles.

### Human Health Risk Assessment

In the study, the Human Health Risk Assessment was performed according to the point estimate method developed by USEPA^68^. In our research, mean and P95 concentration values of the investigated PHEs were used in the calculations (1) for all of Poland and (2) for individual provinces, based on the values obtained from the relevant literature review.

#### Hazard identification

The investigated human health risk was related to the content of PHEs, namely As, Cd, Co, Cu, Hg, Ni, Pb, and Zn, in three groups of edible plants consumed in Poland: vegetables, fruits, and cereals. The PHE contents were obtained from studies conducted in Poland, that aimed to investigate the concentrations of trace elements in edible plants. These results were firstly subjected to statistical characterisation, namely min, max, mean, P95, and SD values were determined, for both Poland and individual provinces (depending on whether the research was performed in these regions before). Due to the too small number of references from Pomerania and Warmia-Masuria provinces, the results from these two regions were grouped as Northern Poland in our studies prior the further analysis. In the risk assessment calculations mean and P95 values for individual PHEs were used.

#### Exposure scenario and exposure pathway

Our study investigated the resident exposure scenario based on the trend that edible plants should be sold and eaten as locally as possible to maintain their freshness and nutritional properties. Moreover, as Poles move reluctantly and instead spend most of their lives in one place^69^, our investigations assumed a lifetime spent in a single geographical location. Regarding edible plants, the investigated exposure pathway assumed consumption by the inhabitants. In this case, apart from the general population, we also considered other subpopulations based on age and sex. The division of these subpopulations was strictly depended on the statistical consumption patterns available from the related studies, namely Nosecka^70^, Łopaciuk^14^, Gheribi^71^, Murawska^72^, Janowska-Miasik et al.^73^, Wolnicka et al.^74^, Zalewska et al.^75^, and Dietary Guidelines for Americans^76^, as well as the recommended daily consumption: WHO^2^, Healthy Eating Plate^3^, National Institute of Public Health (PZH) in Poland^4^, and Australian Dietary Guidelines^77^. Thus, the following subpopulations were distinguished in our investigation based on the available data on the statistical consumption of edible plants in Poland: girls (7–12 years old), boys (7–12 years old), women 18–35 years old, men 18–35 years old, women 36–55 years old, men 36–55 years old, women 56–65 years old, men 56–65 years old, and retirees (> 65 years old). As no statistical data were available for toddlers (1–3 years old), pre-schoolers (4–6 years old), and adolescents (13–18 years old) for Poland, recommended intake values described in the section above were used in the case of these subpopulations. Additionally, the recommended intake values of consumption for school-aged children (7–12 years old) and adults (> 18 years old) were also used in our study. It should be also added that in our investigations potatoes were excluded from the total consumption of vegetables due to their nutritional properties according to Healthy Eating Plate^3^. Moreover, in Poland potato is the basic vegetable consumed and there is the detailed characteristic of their consumption available.

#### Daily intake rate (DIR) and average daily dose (ADD) calculations

The values of the daily intake rate (DIR) for individual PHEs were calculated as the total amount of consumed edible plants from the three edible plant groups, namely vegetables, fruits, and cereals according to the Eq. (1)^78^:

1 $${\text{DIR }} = \, \Sigma \, \left( {{\text{C }} \times {\text{ IR}}/{\text{BW}}} \right)$$

where C is the concentration of the individual PHE in the group of edible plants (mg/kg wet weight; further ww.); IR is the intake rate of a given group of food plants in grams per person per day (g/person-day); BW is the body weight (kg)^78–81^. The intake rate (IR) values of edible plant groups used for risk calculations for the investigated subpopulations are presented in Table 2. Body weight values used for risk calculations are presented in Table 3.

**Table 2:** Intake rate (IR) values (g ww./person-day) of the consumed groups of edible plants used in the study.

Edible plant group	Intake rate (IR) (g ww./person-day)													
	Toddlers	Pre-schoolers	School-aged children	Girls	Boys	Adolescents	Women 18–35	Men 18–35	Women 36–55	Men 36–55	Women 56–65	Men 56–65	Adults	Retirees
All vegetables (ex. potato)	300.0^76^	400.0^76^	440.0^76^	155.2^74^	163.8^74^	500.0^76^	212.0^73^	254.0^73^	245.0^73^	268.0^73^	253.0^73^	281.0^73^	800.0^76^	222.0^73^
Root	39.6^70^	52.8^70^	58.1^70^	20.5^70^	21.6^70^	66.0^70^	28.0^70^	33.5^70^	32.3^70^	35.4^70^	33.4^70^	37.1^70^	105.6^70^	29.3^70^
Leaf	45.0^76^	60.0^76^	66.0^76^	12.9^76^	13.6^76^	75.0^76^	17.6^76^	21.1^76^	20.3^76^	22.2^76^	21.0^76^	23.3^76^	120.0^76^	18.4^76^
Fruit	82.2^70^	109.6^70^	120.6^70^	42.5^70^	44.9^70^	137.0^70^	58.1^70^	69.6^70^	67.1^70^	73.4^70^	69.3^70^	77.0^70^	219.2^70^	60.8^70^
Inflorescence	9.6^70^	12.8^70^	14.1^70^	5.0^70^	5.2^70^	16.0^70^	6.8^70^	8.1^70^	7.8^70^	8.6^70^	8.1^70^	9.0^70^	25.6^70^	7.1^70^
Legume	12.0^71^	14.0^71^	16.0^71^	0.9^71^	1.0^71^	22.0^71^	1.7^71^	1.7^71^	1.7^71^	1.7^71^	1.7^71^	1.7^71^	32.0^71^	2.7^71^
Potato	–	–	–	139.7^72^	147.4^72^	–	177.0^72^	292.0^72^	204.0^72^	281.0^72^	203.0^72^	278.0^72^	–	153.0^72^
All fruits	240.0^75^	250.0^75^	325.0^75^	185.2^74^	194.4^74^	400.0^75^	209.0^73^	164.0^73^	223.0^73^	199.0^73^	246.0^73^	218.0^73^	250.0^75^	151.0^72^
Berry	26.9^70^	28.0^70^	36.4^70^	20.7^70^	21.7^70^	44.8^70^	23.4^70^	18.4^70^	25.0^70^	22.3^70^	27.6^70^	24.4^70^	28.0^70^	18.8^72^
Pome	62.2^70^	64.8^70^	84.2^70^	48.0^70^	50.3^70^	103.6^70^	54.1^70^	42.5^70^	57.8^70^	51.5^70^	63.7^70^	56.5^70^	64.8^70^	50.3^72^
Stone	27.6^70^	28.8^70^	37.4^70^	21.3^70^	22.4^70^	46.0^70^	24.0^70^	18.9^70^	25.6^70^	22.9^70^	28.3^70^	25.1^70^	28.8^70^	17.4^70^
All cereals	175.0^77^	175.0^77^	200.0^77^	–	–	280.0^77^	176.0^73^	282.0^73^	171.0^73^	259.0^73^	160.0^73^	227.0^73^	240.0^77^	228.3^71^
Wheat	64.5^14^	64.5^14^	73.7^14^	–	–	103.1^14^	65.1^14^	104.3^14^	63.3^14^	95.8^14^	59.2^14^	84.0^14^	88.4^14^	84.5^14^
Rye^a^	26.8^14^	26.8^14^	30.7^14^	–	–	42.9^14^	9.8^14^	15.7^14^	9.5^14^	14.4^14^	8.9^14^	12.6^14^	36.8^14^	12.7^14^
Barley	9.9^14^	9.9^14^	22.9^14^	–	–	15.8^14^	19.4^14^	31.0^14^	18.8^14^	28.5^14^	17.6^14^	25.0^14^	13.6^14^	25.1^14^
Oat	1.5^14^	1.5^14^	8.7^14^	–	–	2.4^14^	7.0^14^	11.3^14^	6.8^14^	10.4^14^	6.4^14^	9.1^14^	2.1^14^	9.1^14^

– no data.

^a^Values taken for triticale due to the lack of values for rye.

**Table 3:** Body weight values used in the study for risk calculations.

Subpopulation	Abbreviation	Body weight [kg]	References
Toddlers (1–3 years old)	Toddlers	15	^81^
Pre-schoolers (4–6 years old)	Pre-schoolers	20	^81^
School-aged children (7–12 years old)	School-aged	30	^81^
Girls (7–12 years old)	Girls	30	^81^
Boys (7–12 years old)	Boys	30	^81^
Adolescents (13–18 years old)	Adolescents	55	^81^
Women 18–35 years old	Women 18–35	72	^79^
Men 18–35 years old	Men 18–35	85	^79^
Women 36–55 years old	Women 36–55	76	^79^
Men 36–55 years old	Men 36–55	90	^79^
Women 56–65 years old	Women 56–65	77.5	^79^
Men 56–65 years old	Men 56–65	89.5	^79^
Adults (> 18 years old)	Adults	70	^79^
Retirees (> 65 years old)	Retirees	76	^79^

The Average Daily Dose (ADD) values of individual PHEs resulting from the daily consumption of edible plant groups (mg/kg bw-day) were calculated using the Eq. (2)^68^:

2 $${\text{ADD }} = \, \Sigma \, \left( {{\text{C }} \times {\text{ IR }} \times {\text{ EF }} \times {\text{ ED }} \times { 1}0^{{ - {3}}} } \right)/{\text{AT}} \times {\text{BW}}$$

where C is the PHE concentration in the investigated group of edible plants (mg/kg ww.); IR is the intake rate of edible plants (g ww./person-day); EF is the exposure frequency: 365 days/year; ED is the exposure duration: number of life years in the individual subpopulation; AT is the averaging time in days: ED × 365 for non-carcinogens and 70 years × 365 for carcinogens; BW is body weight (kg), and 10^–3^ is the unit conversion factor^68,82^.

Regarding the IR values used in the studies included both the recommended and statistical daily intake, however not for all subpopulations as both types of data were not available. For instance, statistical values were not available for the most vulnerable groups, i.e., toddlers, pre-schoolers, and adolescents, who also are in the crucial developmental stage of their lives. At least four different literature sources were necessary to obtain the consumption data for a single age group, increasing the risk of occurring bias. Most studies on edible plant consumption focused only on their classification into vegetables, fruits, and cereals, limiting our research to these groups for the most part. The studies we reviewed also differed regarding the time they were conducted, the region, the size of the studied population, and the analytical method of determining PHE contents. Regardless of whether the method considered the amount of food purchased or the consumption declared by the respondents, these data are neither completely reliable nor directly comparable. Moreover, it was not possible to estimate all data points. There were no available data for cereal consumption for boys and girls, thus the calculated risk for those groups is likely lower than in reality.

#### Hazard quotient (HQ) and cancer risk (CR) calculations

The non-carcinogenic risk was determined based on the calculation of the Hazard Quotient (HQ) values (unitless) in accordance with the Eq. (3):

3 $${\text{HQ }} = {\text{ ADD}}/{\text{RfD}}$$

where ADD is the average daily dose (mg/kg bw-day) and RfD is the reference dose (mg/kg bw-day)^68^.

The total non-carcinogenic risk (HQ_t_) value for the investigated PHEs was calculated, using the Eq. (4):

4 $${\text{HQ}}_{{\text{t}}} = {\text{ HQ}}_{{1}} + {\text{ HQ}}_{{2}} + \, \cdots \, + {\text{ HQn}}$$

where HQs are the hazard quotient values for 1−n PHEs investigated in the study.

The carcinogenic risk was determined based on the Cancer Risk (CR) values (unitless) calculations using Eq. (5):

5 $${\text{CR }} = {\text{ ADD}} \times {\text{SF}}_{{\text{o}}}$$

where CR is the carcinogenic risk and SF_o_ is the oral slope factor ((mg/kg bw-day)^−1^) for an individual PHE. In our studies, only As was considered as a carcinogenic PHE due to the lack of SF values in the toxicological databases for other trace elements. The total carcinogenic risk value, as the sum of partial CR values, was not calculated since As was the only carcinogenic PHE considered in this study.

As there is no agreement on the RfD values in the case of Pb in our research, we have also used the margin of exposure (MOE) method, in line with the recommendations of the European Food Safety Authority^83^ in accordance with the risk resulting from the Pb exposure in consumed edible plants based on the Eq. (6):

6 $${\text{MOE }} = {\text{ BMDL}}/{\text{DIR}}$$

where MOE is the margin of the exposure value; BMDL is the benchmark dose (lower confidence limit) and the DIR is the total amount of edible plants consumed daily in the investigated subpopulations.

Based on the information available in the toxicological databases, in our research for As, Cd, Co, Cu, Hg, Ni, Pb, and Zn, the non-carcinogenic risk (HQ) was calculated regarding the toxicological influence of these elements on organisms. Based on the information available on carcinogenic effects, the carcinogenic risk (CR) was only calculated for As in the study. The following reference dose values (RfD) (mg/kg bw-day) were used for the non-carcinogenic risk (HQ) calculations: As 3.0 × 10^–4^, Cd 1.0 × 10^–4^, Co 3.0 × 10^–4^, Cu 4.0 × 10^–2^, Hg 3.0 × 10^–4^, Ni 2.0 × 10^–2^, Pb 1.5 × 10^–3^ and Zn 3.0 × 10^–1^^84^. For As, the oral slope factor (SF_o_) value was equal to 1.5 (mg/kg bw-day)^−1^^84^. In the case of Pb, for which there is no unanimity regarding the RfD value, the calculations based on BMDL estimated at 1.2 μg/kg bw-day for adults and 0.6 μg/kg bw-day for children were also used^83^.

#### Risk characterisation

A risk characterisation was performed using the PHE content collected from literature research for Poland and for individual provinces based on the recommended edible plant consumption doses in the investigated subpopulations. As the acceptable non-carcinogenic risk, the values of the calculated hazard quotient ≤ 1 (HQ ≤ 1) were set, both for individual PHEs (HQ_s_), as well as for the total hazard quotient (HQ_total_), defined as the sum of partial HQ values for individual PHEs^68,85,86^. For carcinogenic risk (CR), the acceptable risk level was set below the value of 1 × 10^–5^, based on the *Regulation of the Minister of the Environment of September 1, 2016 on the method of assessing the pollution of the earth's surface*^86^.

## Results

### Contents of PHEs in edible plants in Poland

Based on the geographical locations of the research in the analysed 27 articles, it was observed that most of the studies were performed in the south of Poland (Fig. 2). This region of Poland was intensively used in the past due to the exploitation and processing of coal and metal ores^87,88^ causing the contamination of the southern Polish region environment with metals like Pb, Zn, Cu, As, Tl, and Cd^89–93^, which resulted in the intensive investigation of metal contents in the water–soil–plant system. On the other hand, in the other regions of Poland where industrial activities were not intensively practised, the number of investigations was significantly lower. The summary statistics of the results of PHEs contents in edible plants collected from the literature research for all of Poland is presented in Table 4. For the individual provinces, a similar summary is presented in Supplementary Table S2. Based on low availability of data and their low contents in edible plants, Sb and Tl were excluded from further analysis in the study. The results gathered revealed that the concentration of the investigated PHEs in edible plants varied from below the level of detection (LOD) to significant level, which was especially observed in leafy plants and edible roots. Considering the mean PHE contents, it was observed that the concentration of elements in all edible plants was in the following decreasing order: Zn > Pb > Cu > Ni > Cd > Hg > As > Co. For vegetables, fruits, and cereals, the concentration of elements was in the following decreasing orders, respectively: Zn > Cu > Pb > Cd > Ni > Hg > As > Co, Zn > Cu > Ni > Pb > Cd > Co, and Pb > Cd > Hg.

**Table 4:** Summary statistics for PHE contents in edible plants from the literature research for all of Poland; – not available; data gathered from articles verified during the literature review presented in Supplementary Table S1.

PHEs	Poland							
	As	Cd	Co	Cu	Hg	Ni	Pb	Zn
Plants	min–max; mean; P95 (mg/kg)							
Vegetables								
Leaf	–	0.002–0.633; 0.083; 0.30	0.000025–0.047; 0.010; 0.049	0.005–33.33; 3.10; 9.08	0.00005–0.171; 0.043; 0.137	–	0.000025–1.94; 0.17; 0.43	0.87–115; 17.71; 91.89
Fruit	0.0034–0.032; 0.014; 0.027	0.0008–0.032; 0.010; 0.024	0.000025–0.005; 0.002; 0.004	0.298–0.413; 0.34; 0.41	0.00007–0.001; 0.0006; 0.001	0.109–0.237; 0.14; 0.21	0.000025–0.25; 0.06; 0.17	0.82–3.50; 2.05; 3.49
Inflorescence	0.00005–0.041; 0.0063; 0.039	0.005–0.065; 0.015; 0.047	0.000025–0.005; 0.003; 0.005	0.50–0.94; 0.72; 0.92	–	–	0.04–0.17; 0.08; 0.14	5.22–9.29; 7.26; 9.09
Bean/pod	–	0.002–0.012; 0.007; 0.011	0.000025–0.035; 0.018; 0.035	0.128–15.32; 6.63; 13.92	–	–	0.033–0.13; 0.08; 0.12	2.91–104.5; 37.4; 94.5
Root	0.00005–0.041; 0.0063; 0.039	0.000025–6.12; 0.66; 2.6	0.000025–0.047; 0.013; 0.043	0.032–5.7; 1.1; 5.0	0.00005–0.167; 0.037; 0.148	0.005–0.24; 0.05; 0.20	0.000025–0.25; 0.06; 0.17	0.82–3.5; 2.05; 3.49
Potato	–	0.005–1.7; 0.26; 1.1	–	0.27–0.56; 0.42; 0.55	–	–	0.019–0.07; 0.03; 0.05	2.09–2.24; 2.17; 2.23
Fruits								
Berry	–	0.00005–0.081; 0.038; 0.068	0.00005–0.014; 0.007; 0.014	0.006–2.30; 0.55; 1.48	–	0.02–0.1; 0.08; 0.1	0.00005–0.69; 0.07; 0.25	0.72–15; 5.36; 13.44
Pome	–	0.00005–0.116; 0.028; 0.110	0.00005–0.17; 0.061; 0.15	0,00–2.29; 0.43; 2.03	–	0.001–2.23; 0.11; 0.2	0.00005–0.33; 0.07; 0.32	0.20–5.34; 0.79; 1.4
Stone	–	0.0–0.069; 0.009; 0.06	0.00005–0.003; 0.001; 0.003	0.0–2.75; 0.83; 2.51	–	0.04–0.3; 0.12; 0.25	0.0–1.57; 0.23; 1.35	0.20–3.03; 1.32; 2.69
Cereals								
Wheat	–	0.00005–0.043; 0.026; 0.042	–	–	0.00005–0.08; 0.032; 0.072	–	0.000025–6.76; 1.14; 6.15	–
Rye	–	–	–	–	–	–	0.000025–6.01; 2.14; 5.92	–
Barley	–	0.003–0.291; 0.114; 0.284	–	–	–	–	0.000025–6.52; 1.53; 6.23	–
Oat	–	–	–	–	–	–	0.000025–12.0; 4.27; 11.82	–

### Risk assessment

Provisional tolerable daily intake (PTDI) values and suggested amounts to be consumed placed on food products are only recommendations and refer to the daily intake without considering the total diet and long-term health impact results. Thus, in our studies, we applied a risk assessment approach to evaluate if the content of PHEs present in edible plants and consumed plant amounts based on consumption questionnaires may pose a threat to the health of Polish consumers. For all the investigated PHEs (As, Cd, Co Cu, Hg, Ni, Pb, and Zn), the non-carcinogenic risk values were calculated since all of them reveal toxic properties. Some of them are also considered carcinogenic. However, due to the lack of required information from the toxicological databases, the carcinogenic risk assessment was performed only for As. As mentioned before, due to the majority of results being below the limit of detection for Sb and Tl, these two PHEs were excluded from the risk analyses.

#### Non-carcinogenic risk

**Figure 3:**
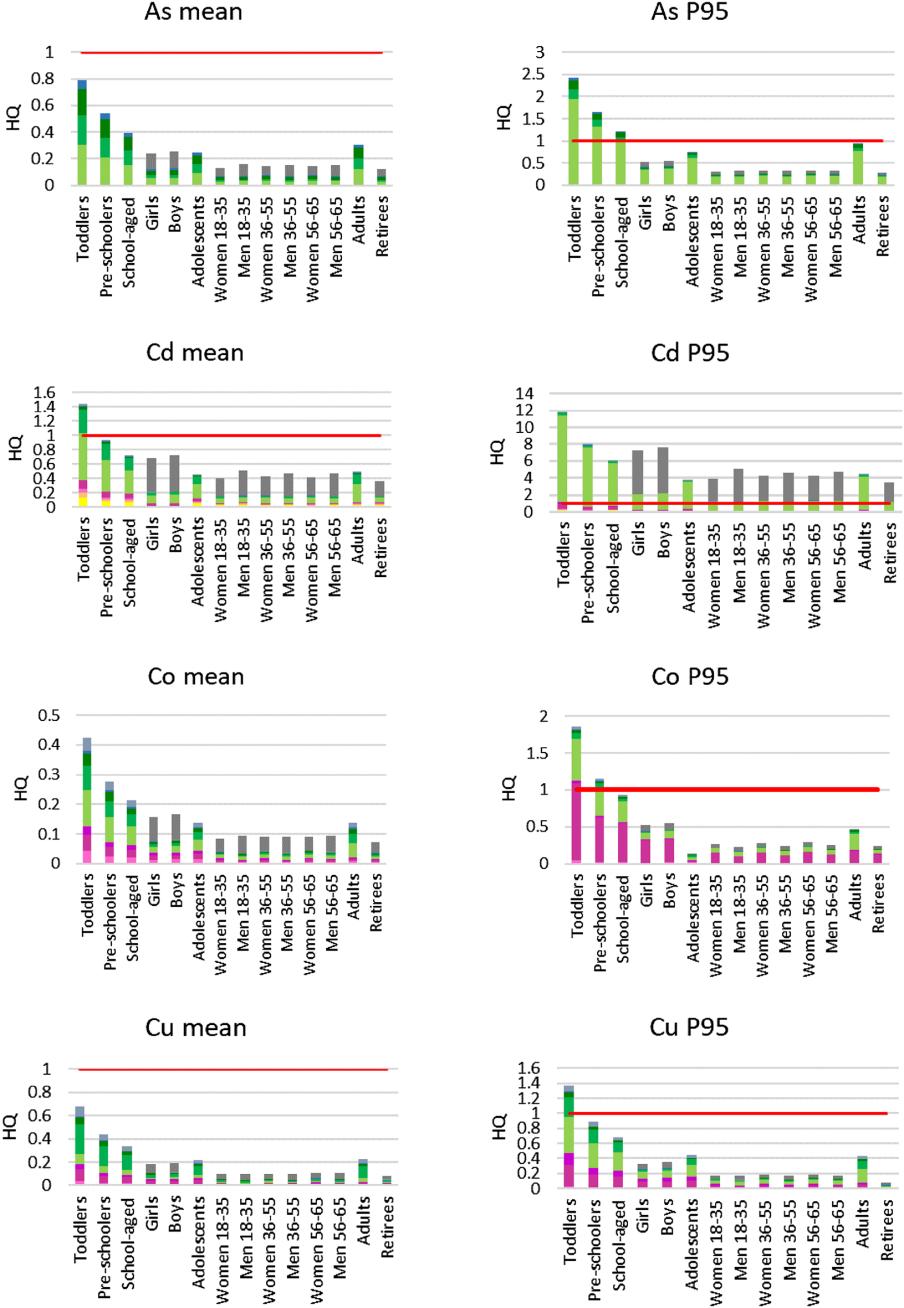

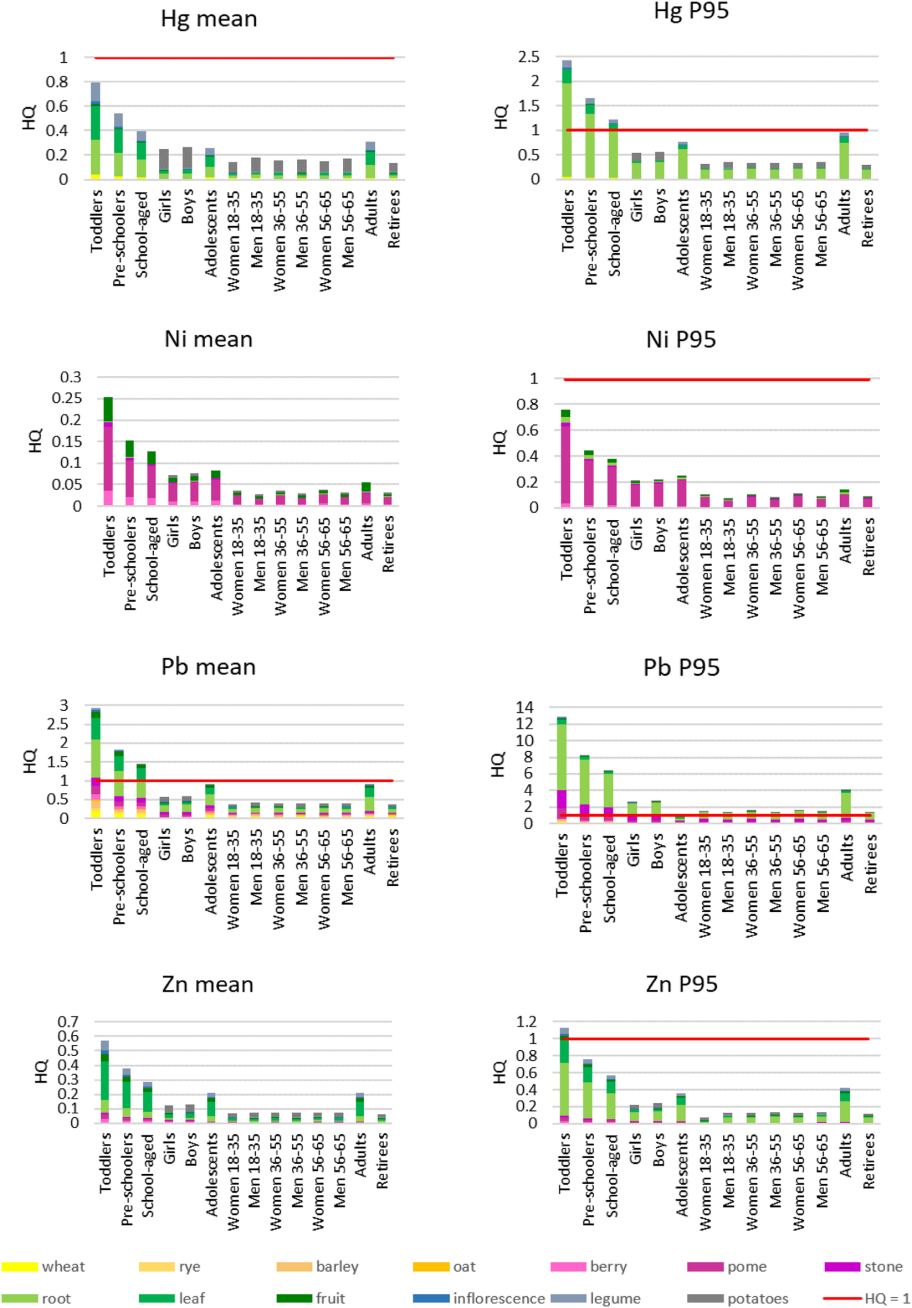
Total non-carcinogenic risk (HQ_total_) values for consumed edible plants in Poland based on mean and P95 concentrations of PHEs; P95—95th percentile.

To calculate the values of hazard quotient (HQ) that describe the non-carcinogenic risk, firstly, the average daily dose (ADD) values were calculated based on the recommended intake of individual edible plants depending on age and sex among subpopulations. Also, the ADD values were calculated in reference to mean and P95 concentrations in edible plants, as well as for Poland and various provinces separately. Next, the results of ADD values were used to calculate the hazard quotient (HQ) values, as well as the total non-carcinogenic risk as the sum of separate HQ values. HQ values calculated for mean and P95 values for PHEs in Poland are presented in Fig. 3, and for individual provinces in Supplementary Fig. S1.

Regarding the mean concentrations calculated from the available results for all of Poland, the highest unacceptable non-carcinogenic risk values for mean PHE contents were found for Pb for toddlers (2.80), pre-schoolers (1.80), and school-aged children (1.45), while in the case of adolescents and adults the risk value was almost at the level of 1. For Cd, the level of 1 was exceeded for toddlers (1.42), and in the case of pre-school children was close to the acceptable level. In the case of the 95th percentile data (P95), the acceptable non-carcinogenic risk value was not exceeded only in the case of Ni for all investigated subgroups. For P95 Pb contents, the non-carcinogenic risk was exceeded for toddlers: As (2.40), Co (2.80), Cu (1.35), Hg (2.40), Zn (1.10), pre-schoolers: As (1.60), Co (1.10), Hg (1.60), school-aged children: As (1.20) and Hg (1.20). For Cd and Pb, the acceptable risk was exceeded for all subpopulations and their highest HQ values were found for toddlers (Cd 12.0 and Pb 12.5).

For all analysed PHEs, the general trend was observed that the most susceptible were the subpopulations of various aged children. For the 95th percentile (P95) contents of PHEs in edible plants, their share in the risk value differed as follows. For As in the real intake for girls, boys, women, and men 18–35, women and men 36–55, women and men 56–65, and retirees, other edible roots had a higher contribution than potatoes. For Co pome fruits, not-root plants, and potatoes had the highest contribution. For Cu, Hg, Pb, and Zn root plants’ contribution increased significantly compared to their mean values in these plants.

Regarding the individual provinces (Supplementary Fig. S1), the highest risk values were observed in the Silesia Province for toddlers > pre-schoolers > school-aged children > adults > adolescents > boys > girls > women 56–65 > women 36–55 > men 56–65 > retirees > men 18–35 > men 36–55 > women 18–35 for Cd > Pb > Cu > As > Hg > Zn > Ni > Co. The highest risk was 4.44 for mean and 19.74 for P95 Cd concentrations for toddlers. In the following provinces, acceptable risk was also exceeded: Lower Silesia for Pb, Lublin for Cd, Cu, Pb, Zn, Lesser Poland for As, Cd, Co, Cu, Hg, Pb, Opole for Hg, Pb, and Northern Poland for Pb. Risk values were the lowest in the Northern Poland, where the highest risk and only exceedance (0.295) was observed for toddlers for P95 of the Pb contents and following decreasing order for investigated PHEs contributing to the overall risk was found: Pb > Cd > Cu > Zn > Co > Ni. In most of studied provinces, the decreasing order of risk values for Pb was as follows: toddlers > pre-schoolers > school-aged children > adolescents and adults > boys and girls > other investigated subpopulations. Only in Northern Poland risks for boys and girls exceeded those of adolescents and adults.

#### Carcinogenic risk

**Figure 4:**
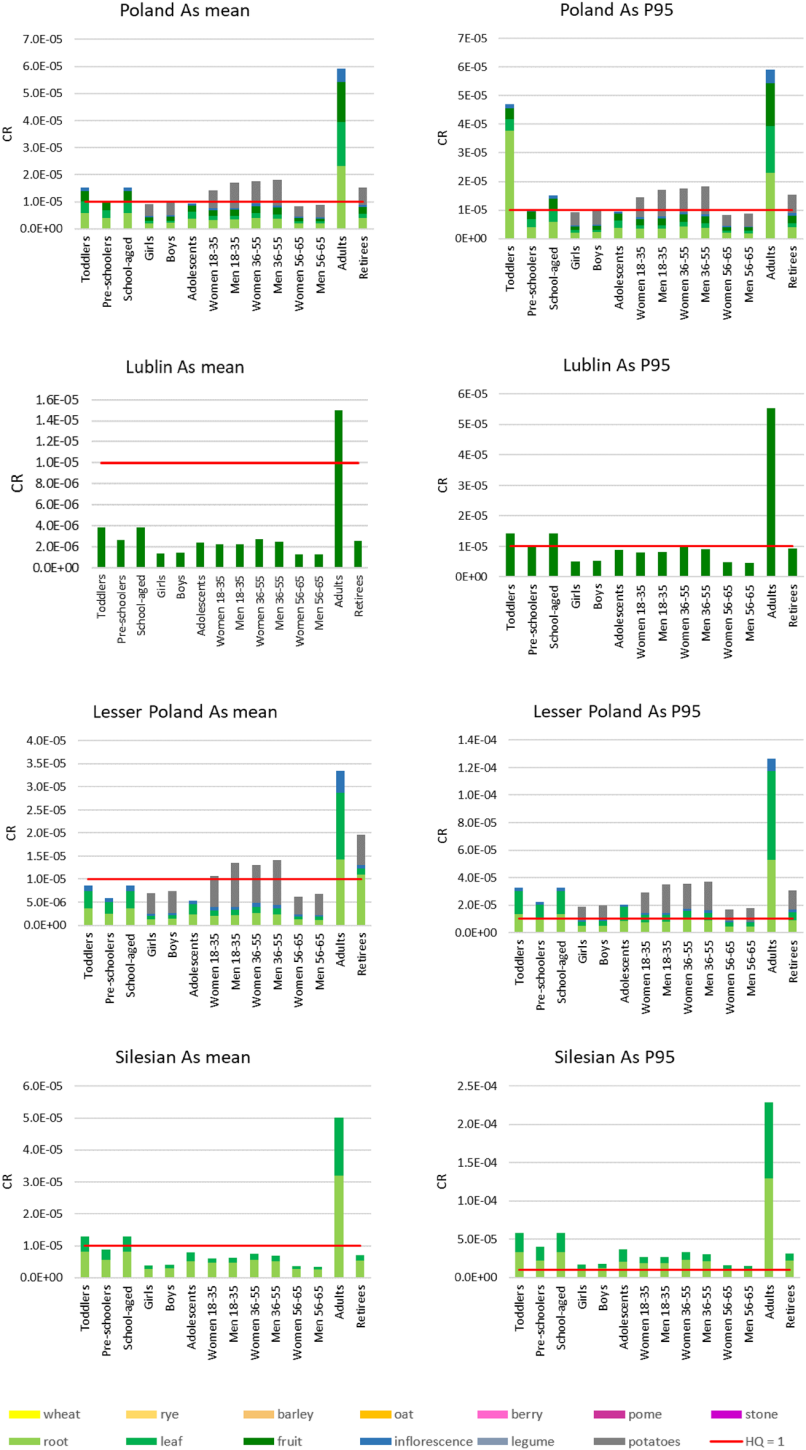
Total carcinogenic risk (CR_total_) values for consumed edible plants in Poland and individual provinces based on mean and P95 concentrations of As; P95—95th percentile.

In the case of the carcinogenic risk, ADD values were also used to calculate the CR values. The CR values calculated for mean and P95 values for PHE contents for all of Poland and in individual provinces are presented in Fig. 4. The calculated carcinogenic risk values for all of Poland revealed a non-acceptable risk for all investigated subpopulations both for mean and P95 values of As in edible plants, except for the women and men 56–65 and girl subpopulations. However, these values approached the acceptable level of 1 × 10^–5^. The highest risk values were observed for adults for mean (5.9 × 10^–5^) and P95 (1.8 × 10^–4^) As contents. The decreasing order of the CR risk from As consumption with particular plant types was as follows: root > leaf > fruit > inflorescence.

Considering individual provinces, it was observed that the acceptable carcinogenic risk level was exceeded in the Lublin province for adults for mean As contents and for toddlers and school-aged for P95 As contents. In the Lesser Poland province, the CR risk level was exceeded for adults, retirees, men 36–55, women and men 18–35, women and men 36–55 for mean As contents and for P95 As contents for all subpopulations in the following decreasing order: adults > men 36–55 > women 36–55 > men 18–35, toddlers > school-aged children > retirees > pre-schoolers > adolescents > boys > girls > men 56–65 > women 56–65. In the Silesia Province, the CR risk was exceeded for adults, toddlers, and school-aged for mean As contents, as well as for P95 As contents for all subpopulations with the following decreasing order: adults > toddlers > school-aged children > pre-schoolers > adolescents > women 36–55 > retirees > men 36–55 > men 18–35 > women 18–35 > boys > girls > women 56–65 > men 56–65.

#### MOE approach for Pb

The Margin of Exposure (MOE) approach was used in our study for Pb, as well as other toxicological values which were not available in the databases. The MOE values were calculated for mean and P95 concentrations of Pb in Poland and are presented in Table 5 and for individual provinces in Supplementary Table S3.

**Table 5:** Margin of exposure (MOE) values for mean and P95 concentrations of Pb in edible plants consumed in Poland; – lack of data; **value** < 1 indicates high health risk; calculated based on articles verified during the literature review presented in Supplementary Table S1.

Poland mean															
		Toddlers	Pre- schoolers	School-aged children	Girls	Boys	Adolescents	Women 18–35	Men 18–35	Women 36–55	Men 36–55	Women 56–65	Men 56–65	Adults	Retirees
Vegetables	Root	**0.40**	**0.59**	**0.80**	2.28	2.16	1.30	8.00	7.89	7.31	7.91	7.22	7.51	2.06	8.07
	Leaf	**0.69**	1.02	1.39	7.11	6.74	2.24	25.00	24.63	22.83	24.72	22.55	23.44	3.56	25.20
	Fruit	2.34	3.43	4.68	13.27	12.57	7.55	46.63	45.94	42.59	46.11	42.06	43.73	12.01	47.00
	Inflorescence	11.95	17.52	23.90	67.74	64.19	38.55	238.05	234.56	217.43	235.39	214.71	223.25	61.33	239.96
	Legume	7.42	11.30	14.83	252.72	239.46	19.77	670.02	790.99	707.24	837.52	721.20	832.87	34.61	445.30
	Potatoes	–	–	–	3.01	2.85	–	11.40	8.16	10.44	8.98	10.70	9.02	–	13.92
Fruits	Berry	2.80	4.86	5.61	9.84	9.37	8.35	41.84	62.95	41.39	54.93	38.26	49.86	34.01	54.99
	Pome	1.79	3.11	3.58	6.29	5.99	5.34	26.76	40.26	26.47	35.13	24.47	31.89	21.73	30.40
	Stone	1.76	3.05	3.52	6.18	5.89	5.25	26.28	39.55	26.00	34.51	24.04	31.32	21.33	38.40
Cereals	Wheat	1.97	3.00	3.94	–	–	5.16	21.40	15.77	23.25	18.18	25.34	20.62	15.33	17.41
	Rye	4.89	7.46	9.77	–	–	12.82	147.42	108.62	160.16	125.22	174.55	142.08	38.04	119.96
	Barley	2.62	8.10	5.24	–	–	13.92	29.75	21.92	32.32	25.27	35.23	28.67	41.18	24.21
	Oat	6.09	46.60	12.17	–	–	80.88	72.19	53.19	78.43	61.32	85.48	69.58	235.29	58.75

Poland P95															
		Toddlers	Pre- schoolers	School-aged	Girls	Boys	Adolescents	Women 18–35	Men 18–35	Women 36–55	Men 36–55	Women 56–65	Men 56–65	Adults	Retirees
Vegetables	Root	**0.05**	**0.59**	**0.10**	**0.29**	**0.27**	**0.16**	1.01	**1.00**	**0.92**	1.00	**0.91**	**0.95**	**0.26**	1.02
	Leaf	**0.69**	1.02	1.39	7.11	6.74	2.24	25.00	24.63	22.83	24.72	22.55	23.44	3.56	25.20
	Fruit	2.34	3.43	4.68	13.27	12.57	7.55	46.63	45.94	42.59	46.11	42.06	43.73	12.01	47.00
	Inflorescence	11.95	17.52	23.90	67.74	64.19	38.55	238.05	234.56	217.43	235.39	214.71	223.25	61.33	239.96
	Legume	7.42	11.30	14.83	252.72	239.46	19.77	670.02	790.99	707.24	837.52	721.20	832.87	34.61	445.30
	Potatoes	–	–	–	3.01	2.85	–	11.40	8.16	10.44	8.98	10.70	9.02	–	13.92
Fruits	Berry	2.80	4.86	5.61	9.84	9.37	8.35	41.84	62.95	41.39	54.93	38.26	49.86	34.01	54.99
	Pome	**0.35**	3.11	**0.69**	1.21	1.16	1.03	5.17	7.77	5.11	6.78	4.72	6.16	4.20	5.87
	Stone	**0.18**	3.05	**0.36**	**0.62**	**0.59**	**0.53**	2.65	3.99	2.63	3.49	2.43	3.16	2.15	3.88
Cereals	Wheat	1.97	3.00	3.94	–	–	5.16	21.40	15.77	23.25	18.18	25.34	20.62	15.33	17.41
	Rye	4.89	7.46	9.77	–	–	12.82	147.42	108.62	160.16	125.22	174.55	142.08	38.04	119.96
	Barley	2.62	8.10	5.24	–	–	13.92	29.75	21.92	32.32	25.27	35.23	28.67	41.18	24.21
	Oat	6.09	46.60	12.17	–	–	80.88	72.19	53.19	78.43	61.32	85.48	69.58	235.29	58.75

Considering the MOE values for all of Poland the high health risk (MOE values < 1) was indicated for mean Pb contents with leaf and root plants consumption for toddlers, and root plant consumption for school-aged children and pre-schoolers. For P95 contents of Pb, the MOE values were < 1 for all investigated subpopulations, except women 18–35, men 36–55 and retirees, with the highest risk observed for the consumption of root, stone, pome, and leaf plants.

Considering the individual provinces, the unacceptable risk was observed in the Lower Silesia Province for leaf plants consumption by toddlers, pre-schoolers, school-aged children, and adults for both mean and P95 Pb values, as well as for adolescents for P95 Pb contents. In the Lublin province, the unacceptable risk was stated for mean Pb contents for root plants for pre-schoolers, school-aged children, adults, and adolescents and for P95 Pb values for P95 Pb values for root, leaf, and fruit plants for toddlers and pre-schoolers, for root and leaf plants for school-aged children, adults, and adolescents, and for root plants for girls and boys. In the Lesser Poland province, the unacceptable risk was determined for mean Pb contents for leaf and stone plants for toddlers and pre-schoolers, for leaf plants for school-aged children and adults and for P95 Pb contents for leaf, stone, and fruit plants for toddlers, pre-schoolers, and school-aged children, for stone and leaf plants for adolescents, girls, and boys, and for leaf and fruit plants for adults. In the Opole province, unacceptable risk level was found only for P95 Pb contents for berry plants for toddlers, pre-schoolers, and school-aged children. In the Silesia Province, unacceptable risk level was found for P95 Pb contents for root and pome plants for toddlers and for root plants for pre-schoolers, school-aged children, adults, adolescents, boys, and girls. In Northern Poland, the calculated MOE values were > 1, indicating no risk to consumers from Pb content.

#### Comparison with related studies on risk assessment due to PHEs in edible plants

The results of other research studies on the health risk for consumers related to the consumption of edible plants are in line with our findings. Regarding the available publications from Europe on health risk related with PHEs present in food crops, in the study conducted in Romania^94^ which also suggests unacceptable health risks related to heavy metal intake from vegetables consumption. Since Carpathian Mountains represent a rich source of heavy metals for East European countries, this study allows to confirm that in a region with similar geochemistry, geology, and mining history of that of southern Poland, risk can also be unacceptable with Pb in root vegetables being mainly responsible for that. High risk values were also noted for Pb and Cd in leafy vegetables, which is also in line with our findings. However, this study focuses on individual vegetables, so the overall dietary risk is not presented and therefore cannot be compared. Study on heavy metals in agricultural soils of EU^95^ presents some concerning results when considering our study. Specifically, the study found that heavy metal contents in most regions of Western and Southern Europe were higher than those observed in Poland. However, health risk assessment was not a part of this study and therefore there is no information on risk values. The total health risk calculated by Wang et al.^96^ in EU28 for corn ingestion were equal to 3.74 × 10^–6^ for adults and 2.08 × 10^–6^ for children and for wheat ingestion were equal to 5.80 × 10^–5^ for adults and 4.30 × 10^–5^ for children^96^. For heavy metal contents in vegetables data can be found for Latvian onion and carrot^97^, with higher contents of Ni (0.25 mg/kg and 0.28 mg/kg, respectively) and Pb (0.09 mg/kg and 0.12 mg/kg, respectively) compared to our study (mean values: 0.05 mg/kg and 0.06 mg/kg, respectively). Cd content reported in Latvia (0.05 mg/kg and 0.12 mg/kg) was considerably higher than in Poland (0.66 mg/kg). However, the aforementioned study only considered onions and carrot, while our results came from the group of root vegetables.

Studies conducted in Nigeria on the consumption of edible plants grown on arable soils in the vicinity of lead and zinc mines reported values of the total hazard quotient in the ingestion of edible plants pathway higher for children than for adults^26^. The risk was higher than acceptable for Cd, Cr, and Pb, with the highest risk concerning Pb for children (2.04) and it was comparable to the risk for children consuming the recommended number of plants (mean of toddlers, pre-schoolers, and school-aged children HQ) for mean Pb values in Poland (2.07). For the P95 Pb values we report here for plant consumption among boys (2.77) and girls (2.63), the HQ exceed the one observed in Nigeria. Research on the health risks of consuming food crops grown near a landfill in Thailand^98^, showed very high HQ for As (47.28), while the highest HQ for P95 As values in Poland for toddlers was 2.43 and for none of the subpopulations of adults, the risk exceeded the acceptable limit. Similarly, the carcinogenic risk was also much higher in the Ruchuwararak et al.^98^ study. HQ for Cd observed in Thailand were also higher than the values observed in Poland, although this difference was less sticking. However, the risk values for Pb and Zn were much lower than for Poland. The highest HQ value noted in Thailand was 0.255, well below the lowest risk for mean Pb values we report here for retirees (0.37). Other studies revealed high HQ values for As in all age groups^99–101^, while in this study, HQ > 1 was observed only for children. Other research studies also pointed out that the higher environmental pollution is, the higher PHE content in edible plants is and so is the risk from consumption^102–104^. It is most visible in the study of Cai et al.^105^, where an area polluted by a large Cu-smelter in central China was compared against a reference non-polluted area. The contents of Cd, Cu, Pb, and As in edible plants were significantly higher in the polluted area, and HQ was higher in the affected than in the reference area, ranging from 237% for Pb to 2747% for Cd. We note that this study considered not only edible plants, but also fish and drinking water, however crop intake was a source of 78% of hazardous elements. Similarly, in the study of Yang et al.^99^, contaminations of vegetables and the health risk were both significantly lower for the reference site than for the polluted area.

## Discussion

Generally, it was observed that a higher consumption of edible plants resulted in higher risk values and vegetable, fruit and cereal consumption was lower than recommended for healthy living. Although higher intake was related to higher risk values, it is important to consider that lower intakes may also be considered unhealthy from a dietary perspective. Based on the trend that risk was higher while considering the recommended daily intake, it would be valuable to generate robust data for the groups with the highest risk (toddlers and pre-schoolers), in order to confidently conclude whether the risk for these groups is low enough to be considered acceptable. Nevertheless, most of our research was performed on the general human population, and there is very little differentiation between adults and children, and studies rarely consider other subpopulations. Even if data from men, women, and children are collected, they are all grouped together, and the summary statistics used do not differentiate between these subpopulations. Apart from that such an approach yields non-representative results, the underlying assumptions made when grouping the data result in highly uncertain risk characterisations. Moreover, the risk calculation approach based on the recommended consumption rates resulted in the worst-case scenario in terms of the amount of daily intake. A comparison of the PHE contents to the provisional maximum tolerable daily intake (PMDTI) values refers to daily consumption and it focuses more on the nutritional elements in food. In the case of elements conferring adverse health effects, apart from the dose, a critical tool for assessing the impact on human health is the risk assessment procedure. Based on that, the values of non-carcinogenic (HQ) and cancerogenic (CR) risks were calculated for the mean and P95 values of PHEs in Poland obtained from our literature research.

As Poland is diversified regarding its geochemical background and anthropogenic activities, simple mean values of PHEs concentrations from all regions of Poland are not considered as the best approach and were mainly used as a first step in our approach for comparison reasons. To include the impact of the changing nature of geochemical background concentrations, as well as differences in the concentrations of PHEs in edible plants depending on Polish regions, the mean and P95 values calculated for the individual provinces were also used in our risk assessment. Our results suggest that using only mean values may lead to erroneous conclusions, which is extremely important if administrative (i.e., risk management) decisions are made based on these results, for example, on remediation actions. Comparing the results for individual provinces, the highest risk values were observed where the highest contents in plants also occurred. Thus, calculating the risk for the broader region by averaging the results from areas with lower and higher PHE contents might distort the results. However, we assumed an identical consumption level across the regions which is not realistic. Thus, further research is recommended to account for the variability of consumption among the subpopulations in various regions of the country.

Currently the food safety in EU is regulated by a number of law acts with Commission Regulation (EC) No 1881/2006 of 19 December 2006 setting maximum levels for certain contaminants in foodstuffs^106^ being particularly important regarding our research. Also, food safety is strongly supported by the EU agenda European Food Safety Authority (EFSA), established by Regulation (EC) No 178/2002 of the European Parliament and of the Council of 28 January 2002 laying down the general principles and requirements of food law^107^. Additionally, in Poland food safety is controlled by the Chief Sanitary Inspectorate^108^. Regarding the consumer safety, the risk values calculated in our research were overestimated based on the used in the research intake rates. Nevertheless, based on the progressive environmental pollution and increasing number of studies reporting presence of PHEs in consumed edible plants, it is recommended to monitor the PHE contents in edible plants as they might indicate a major health issue for their consumers in the nearest future. In our studies, we tried to achieve a more reliable approach, including more consumer groups and using the results of consumption from questionnaire surveys. However, as variability and uncertainty analyses are required for the most reliable results, statistical modelling is recommended in further analysis, especially since our preliminary results indicate that PHE contents in edible plants represent a potential risk to consumers.

## Conclusions

We performed a consumer health risk assessment, based on a comprehensive literature research on the PHE content in edible plants in Poland. For all of Poland, considering the mean PHE contents, the acceptable non-carcinogenic risk level of 1 was exceeded for Pb for toddlers, pre-schoolers, and school-aged children. For Cd, the acceptable risk was exceeded for toddlers and close to the acceptable value for pre-school children. In the case of the 95th percentile of the PHE content, the acceptable non-carcinogenic risk value was not exceeded only in the case of Ni for all investigated subpopulations. The carcinogenic risk values for all of Poland revealed a non-acceptable risk for all investigated subpopulations, both for mean and P95 contents of As in edible plants, except the women and men aged 56–65 and the girl subpopulations. Acceptable level of the non-carcinogenic risk for consumers was exceeded in Silesia, Lower Silesia, Lublin, Lesser Poland, and Opole provinces and of the carcinogenic risk was exceeded in Lesser Poland, Lublin, and Silesia provinces, indicating the impact of geochemical variability on risk assessment results. It is recommended to calculate the risk regionally as national averages do not necessarily represent reality and might lead to erroneous risk characterisations, which can be particularly detrimental when risk management decisions are made based on risk assessment.

## Supplementary Information

Supplementary Information.

## Data Availability

The datasets used and/or analysed during the current study are available from the corresponding author A.G.-K on reasonable request.
