# Supplementary material for: Dietary exposure to potentially harmful elements in edible plants in Poland and the health risk dynamics related to their geochemical differentiation
Source: Sci Rep. 2023 May 25;13:8521. doi: 10.1038/s41598-023-35647-x (PMC10212926; doi:10.1038/s41598-023-35647-x)
Supplement: Supplementary file 1 — Supplementary Information. [file 41598_2023_35647_MOESM1_ESM.pdf]

Dietary exposure to potentially harmful elements in edible plants in Poland and the health risk dynamics related to their geochemical differentiation

Supplementary Information

Agata Wódkowska[1], Agnieszka Gruszecka-Kosowska[1*]

[1]AGH University of Science and Technology, Faculty of Geology, Geophysics, and Environmental Protection, Department of Environmental Protection, Al. Mickiewicza 30, 30-059 Kraków, Poland; awodkows@agh.edu.pl (A.W.), agnieszka.gruszecka@agh.edu.pl (A.G.-K.), [*] corresponding author

Supplementary Table S1. The list of the articles included with PHEs extraction and determination methods in the studies analysed in our research

Supplementary Table S2. Summary statistics of PHE contents in edible plants from the literature review for individual provinces in Poland; - not available

Supplementary Table S3. Margin of exposure (MOE) values for mean and P95 concentrations of Pb in edible plants consumed in individual provinces of Poland; - lack of data; **value** - <1 indicate high health risk

Supplementary Figure S1. Non-carcinogenic risk (HQ) values for consumed edible plants in individual provinces in Poland based on mean and P95 concentrations of PHEs; P95 – 95th percentile

Supplementary Table S1. The list of the articles included with PHEs extraction and determination methods in the studies analysed in our research

Extraction method	Detection Method	Reference
Dry mineralization, 450°C	Atomic Absorption Spectroscopy (AAS)	[1]
Microwave digestion in HNO_3	Inductively Coupled Plasma Optical Emission Spectroscopy (ICP-OES)	[2]
Pressure mineralization in microwave reactor	Inductively Coupled Plasma Mass Spectrometry (ICP-MS)	[3]
Wet mineralization H_2SO_4 + 30% H_2O_2	Atomic Absorption Spectroscopy (AAS)	[4]
Mineralization in H_2SO_4	Atomic Absorption Spectroscopy (AAS)	[5]
Microwave digestion in HNO_3 + H_2O_2	Atomic Absorption Spectroscopy (AAS)	[6]
Dry mineralization at 450°C and HCl digestion	Atomic Absorption Spectroscopy (AAS)	[7]
Digestion in HNO_3 + $HClO_4$ (1:3)	Inductively Coupled Plasma Optical Emission Spectroscopy (ICP-OES)	[8]
Microwave digestion in HNO_3	Inductively Coupled Plasma Optical Emission Spectroscopy (ICP-OES)	[9]
Microwave digestion in HNO_3	Inductively Coupled Plasma Optical Emission Spectroscopy (ICP-OES)	[10]
Pressure mineralisation using microwave energy	Atomic Absorption Spectroscopy (AAS)	[11]
Mineralizer digestion in HNO_3 + H_2O_2 (3:1), 130°C, 2 hours	Inductively Coupled Plasma Mass Spectrometry (ICP-MS)	[12,13,14]
Dry mineralization	Atomic Absorption Spectroscopy (AAS)	[15]
Dry mineralization in 540°C and HCl + HNO_3 digestion	Atomic Absorption Spectroscopy (AAS)	[16]
Dry mineralization in 400°C and HCl + HNO_2 digestion	Atomic Absorption Spectroscopy (AAS)	[17,18]
Dry mineralization in 450°C and HNO_3 + $HClO_4$ digestion	Atomic Absorption Spectroscopy (AAS)	[19]
Dry mineralization and APDC/MBIK extraction method	Atomic Absorption Spectroscopy (AAS)	[20]
Dry mineralization in 450°C and HNO_3 digestion, additionally for Cd and Pb APDC/MBIK extraction	Atomic Absorption Spectroscopy (AAS)	[21]
Microwave digestion in HNO_3	Inductively Coupled Plasma Optical Emission Spectroscopy (ICP-OES), Atomic Absorption Spectroscopy (AAS)	[22,23]
Wet mineralization HCl + HNO_3 (1:3)	Inductively Coupled Plasma Optical Emission Spectroscopy (ICP-OES)	[24]
Digestion in HNO_3 + $HClO_4$ (4:1)	Atomic Absorption Spectroscopy (AAS)	[25]
Pressure microwave wet mineralization in HNO_3	Atomic Absorption Spectroscopy (AAS)	[26]
Dry mineralization in 450°C and HCl digestion	Atomic Absorption Spectroscopy (AAS)	[27]

Supplementary Table S2. Summary statistics of PHE contents in edible plants from the literature research for individual voivodeships in Poland; - not available

**PHEs**	**Lower Silesia**							
	**As**	**Cd**	**Co**	**Cu**	**Hg**	**Ni**	**Pb**	**Zn**
**PLANTS**	**min-max; mean; P95 (mg/kg)**							
**Vegetables**								
Leaf	-	-	-	-	-	-	-	-
Fruit	-	-	-	-	-	-	-	-
Inflorescence	-	-	-	-	-	-	-	-
Bean/pod	-	-	-	-	0.00016-0.0007; 0.00045; 0.00045	-	-	-
Root	-	0.002-0.135; 0.047; 0.108	-	0.005-33.3; 2.55; 5.14	-	-	0.014-1.94; 0.39; 0.49	0.72-2.6; 1.31; 2.4
Potato	-	-	-	-	-	-	-	-
**Fruits**								
Berry	-	-	-	-	-	-	-	-
Pome	-	0.0-0.0024; 0.0008; 0.0020	-	-	-	-	0.0-0.002; 0.0004; 0.002	-
Stone	-	-	-	-	-	-	-	-
**Cereals**								
Wheat	-	-	-	-	-	-	-	-
Rye	-	-	-	-	-	-	-	-
Barley	-	-	-	-	-	-	-	-
Oat	-	0.004-0.015; 0.0095; 0.014	-	0.37-0.64; 0.54; 0.63	-	-	0.011-0.021; 0.013; 0.019	0.87-33.7; 7.8; 21.5
**PHEs**	**Lublin**							
	**As**	**Cd**	**Co**	**Cu**	**Hg**	**Ni**	**Pb**	**Zn**
**PLANTS**	**min-max; mean; P95 (mg/kg)**							
**Vegetables**								
Leaf	-	0.021-0.41; 0.036; 0.36	-	0.56-9.4; 3.66; 8.65	-	-	0.014-0.80; 0.17; 0.53	8.0-115.0; 53.1; 114.5
Fruit	0.003-0.032; 0.007; 0.027	0.005-0.032; 0.009; 0.027	-	0.30-0.41; 0.34; 0.408	0.00007-0.0001; 0.00008; 0.00097	0.11-0.24; 0.14; 0.21	0.008-0.15; 0.025; 0.137	1.63-1.96; 1.85; 1.93
Inflorescence	-	0.011-0.042; 0.026; 0.026	-	0.59-0.92; 0.75; 0.75	-	-	0.011-0.052; 0.028; 0.028	9.97-14.93; 12.11; 12.11

Bean/pod	-	-	-	-	-	-	-	-
Root	-	0.02-0.09; 0.045; 0.081	-	-	-	-	0.15-1.23; 0.69; 1.05	-
Potato	-	-	-	-	-	-	0.019-0.038; 0.026; 0.035	-
**Fruits**								
Berry	-	0.021-0.066; 0.046; 0.065	-	-	-	-	0.009-0.17; 0.041; 0.125	-
Pome	-	-	-	-	-	-	-	-
Stone	-	-	-	-	-	-	-	-
**Cereals**								
Wheat	-	-	-	-	-	-	0.04-0.10; 0.062; 0.098	-
Rye	-	-	-	-	-	-	0.03-0.13; 0.06; 0.123	-
Barley	-	-	-	-	-	-	0.08-0.23; 0.15; 0.22	-
Oat	-	-	-	-	-	-	0.09-0.25; 0.17; 0.24	-
**PHEs**	**Lesser Poland**							
	**As**	**Cd**	**Co**	**Cu**	**Hg**	**Ni**	**Pb**	**Zn**
**PLANTS**	**min-max; mean; P95 (mg/kg)**							
**Vegetables**								
Leaf	0.00005-0.059; 0.013; 0.058	0.006-0.43; 0.13; 0.41	0.002-0.053; 0.013; 0.048	0.104-3.57; 0.78; 2.77	0.00005-0.171; 0.018; 0.089	-	0.000025-2,6; 0.37; 0.18	1.53-39.3; 9.54; 27.8
Fruit		0.004-0.039; 0.006; 0.036	0.001-0.003; 0.0016; 0.0028	0.074-0.64; 0.35; 0.64	-	-	0.000025-0.05; 0.03; 0.05	1.08-3.49; 1.91; 3.19
Inflorescence	0.00005-0.041; 0.021; 0.039	0.011-0.023; 0.017; 0.022	0.001-0.005; 0.003; 0.0048	0.22-0.41; 0.31; 0.40	0.005-0.012; 0.009; 0.012	-	-	2.4-5.5; 3.95; 5.4
Bean/pod		0.005-0.116; 0.039; 0.105	0.0015-0.035; 0.019; 0.0348	0.48-8.9; 2.5; 7.4	0.00005-0.21; 0.04; 0.17	-	0.000025-0.05; 0.03; 0.05	4.4-35.4; 12.4; 30.6
Root	0.00005-0.066; 0.015; 0.055	0.018-0.38; 0.116; 0.32	0.000025-0.047; 0.0097; 0.037	0.102-2.46; 0.66; 1.99	0.00005-0.167; 0.018; 0.089	-	0.000025-2.6; 0.37; 0.18	0.67-14.5; 4.26-11.96
Potato	-	-	-	-	-	-	-	-
**Fruits**								
Berry	-	0.002-0.033; 0.011; 0.029	0.00005-0.014; 0.0058; 0.0058	0.17; 0.74; 0.51; 0.72	-	0.00005-1.5; 0.45; 1.35	0.014-0.24; 0.07; 0.20	-

Pome	-	-	-	-	-	-	-	-
Stone	-	0.00005-0.01; 0.004; 0.009	0.00005-0.003; 0.002; 0.002	0.40-1.12; 0.73; 1.08	-	0.00005-0.024; 0.006; 0.02	0.017-1.57; 0.44; 1.35	-
**Cereals**								
Wheat	-	-	-	-	-	-	-	-
Rye	-	-	-	-	-	-	-	-
Barley	-	-	-	-	-	-	-	-
Oat	-	-	-	-	-	-	-	-
**PHEs**	**Northern Poland**							
	**As**	**Cd**	**Co**	**Cu**	**Hg**	**Ni**	**Pb**	**Zn**
**PLANTS**	**min-max; mean; P95 (mg/kg)**							
**Vegetables**								
Leaf	-	0.055-0.12; 0.089; 0.12	-	-	-	-	0.03-0.13; 0.06; 0.12	-
Fruit	-	0.0008-0.0016; 0.0012; 0.0014	-	-	0.0009-0.0012; 0.0011; 0.0012	-	0.03-0.05; 0.04; 0.049	-
Inflorescence	-	0.005-0.014; 0.009; 0.013	-	-	-	-	0.04-0.17; 0.09; 0.10	-
Bean/pod	-	0.002-0.012; 0.007; 0.011	-	-	-	-	0.03-0.13; 0.075; 0.12	-
Root	-	0.0025-0.041; 0.019; 0.029	-	0.08-1.53; 0.58; 1.29	-	0.005	0.03-0.15; 0.07; 0.11	0.9-2.8; 2.2; 2.8
Potato	-	0.01-0.015; 0.013; 0.015	-	0.27-0.56; 0.42; 0.55	-	0.005	0.03-0.07; 0.05; 0.05	2.09-2.24; 2.17; 2.23
**Fruits**								
Berry	-	-	0.005	0.05-0.8; 0.4; 0.7	-	0.02-0.1; 0.075; 0.1	-	1.2-15.0; 7.5; 13.5
Pome	-	-	0.005	0.0-0.6; 0.3; 0.6	-	0.001-0.2; 0.135; 0.2	-	0.2-1.4; 0.74; 1.4
Stone	-	-	0.005	0.0-0.7; 0.4; 0.7	-	0.04-0.3; 0.12; 0.25	-	0.2-1.5; 0.99; 1.4
**Cereals**								
Wheat	-	0.015-0.04; 0.027; 0.039	-	-	-	-	0.04-0.09; 0.06; 0.087	-
Rye	-	-	-	-	-	-	-	-
Barley	-	0.02-0.064; 0.042; 0.062	-	-	-	-	0.06-0.12; 0.09; 0.116	-

Oat	-	-	-	-	-	-	-	-
**PHEs**	**Opole**							
	**As**	**Cd**	**Co**	**Cu**	**Hg**	**Ni**	**Pb**	**Zn**
**PLANTS**	**min-max; mean; P95 (mg/kg)**							
**Vegetables**								
Leaf	-	0.00003-0.17; 0.035; 0.119	0.000025-0.010; 0.002; 0.008	0.052-1.75; 0.71; 0.49	0.0005-0.125; 0.016; 0.081	-	0.00003-0.59; 0.11; 0.11	2.41-28.0; 6.94; 20.9
Fruit	-	0.00003-0.009; 0.003; 0.009	-	0.06-0.32; 0.17; 0.30	-	-	0.00003-0.02; 0.003; 0.02	-
Inflorescence	-	0.001-0.004; 0.002; 0.003	0.000025-0.0053; 0.0026; 0.0050	0.12-0.18; 0.15; 0.18	-	-	-	3.28-5.11; 4.20; 5.02
Bean/pod	-	0.00003-0.057; 0.013	0.000025-0.0053; 0.0026; 0.0050	0.13-15.3; 3.8; 12.8	-	-	0.00003-0.04; 0.01; 0.04	3.96-104.5; 27.1; 86.3
Root	-	0.001-0.061; 0.018; 0.053	-	0.03-0.71; 0.35; 0.65	0.00005-0.0060; 0.0012; 0.0048	-	0.00003-0.04; 0.01; 0.03	1.67-21.1; 5.72; 17.0
Potato	-	0.002-0.014; 0.006; 0.012	-	0.07-0.23; 0.14, 0.22	-	-	0.00003-0.04; 0.01; 0.04	2.2-5.6; 3.4; 3.4
**Fruits**								
Berry	-	0.00005-0.042; 0.007; 0.031	-	0.15-0.54; 0.36; 0.42	-	-	0.00005-0.54; 0.11; 0.53	2.0-4.4; 3.17; 4.2
Pome	-	-	0.00005-0.0020; 0.0005; 0.0017	0.12-0.56; 0.34; 0.53	-	-	0.00005-0.01; 0.003	0.37-5.3; 2.8; 5.1
Stone	-	0.00005-0.001; 0.0002, 0.0008	-	0.16-1.45; 0.47; 1.20	-	-	0.00005-0.16; 0.07; 0.15	1.15-3.0; 1.9; 2.8
**Cereals**								
Wheat	-	-	-	-	-	-	-	-
Rye	-	-	-	-	-	-	-	-
Barley	-	-	-	-	-	-	-	-
Oat	-	-	-	-	-	-	-	-
**PHEs**	**Silesia**							
	**As**	**Cd**	**Co**	**Cu**	**Hg**	**Ni**	**Pb**	**Zn**
**PLANTS**	**min-max; mean; P95 (mg/kg)**							
**Vegetables**								
Leaf	0.00005-0.16; 0.017; 0.09	0.011-0.63; 0.13; 0.44	0.0-0.009; 0.001; 0.006	0.40-17.3; 4.1; 12.3	0.00005-0.08; 0.02; 0.07	-	0.000025-0.32; 0.07; 0.22	2.2-53.8; 13.8; 43.2
Fruit	-	0.001-0.004;	-	-	-	-	-	-

		0.003; 0.004						
Inflorescence	-	0.02-0.09;<br>0.05; 0.08	-	0.0-1.14;<br>0.54; 0.99	-	-	0.02-0.09;<br>0.05; 0.05	2.8-10.1;<br>7.3; 9.6
Bean/pod	-	-	-	-	-	-	-	-
Root	0.00005-0.17;<br>0.03; 0.13	0.000025-6.1;<br>0.90; 4.4	-	0.10-5.7;<br>1.9; 5.1	0.00005-0.095;<br>0.018; 0.075	-	0.000025-4.4;<br>1.3; 3.7	1.2-60.4;<br>14.8; 49.4
Potato	-	0.005-1.7;<br>0.33; 1.2	-	0.63-2.4;<br>1.3; 2.2	0.011-0.045;<br>0.029; 0.044	0.0001-0.19;<br>0.06; 0.17	0.000025-0.24;<br>0.09; 0.22	1.4-2.4;<br>1.8; 2.3
**Fruits**								
Berry	-	0.00005-0.081;<br>0.036; 0.080	-	0.006-2.30;<br>1.03; 2.27	-	0.00005-1.62;<br>0.38; 1.45	0.00005-0.69;<br>0.21; 0.65	0.99-4.8;<br>2.4; 4.6
Pome	-	0.00005-0.12;<br>0.04; 0.11	-	0.72-2.08;<br>1.60-2.07	-	0.00005-2.23;<br>0.92; 2.06	0.00005-0.33;<br>0.17; 0.31	0.85-3.8;<br>2.0; 3.6
Stone	-	0.00005-0.006;<br>0.002; 0.005	-	0.17-2.8;<br>1.2; 2.5	-	0.00005-0.40;<br>0.10; 0.34	0.00005-0.12;<br>0.03; 0.10	0.64-1.6;<br>1.2; 1.6
**Cereals**								
Wheat	-	-	-	-	-	-	-	-
Rye	-	-	-	-	-	-	-	-
Barley	-	-	-	-	-	-	-	-
Oat	-	-	-	-	-	-	-	-

Supplementary Table S3. Margin of exposure (MOE) values for mean and P95 concentrations of Pb in edible plants consumed in individual voivodeships of Poland; - lack of data; **value** if <1 indicates high health risk

		Toddlers	Pre-schoolers	School-aged	Girls	Boys	Adolescents	Women 18-35	Men 18-35	Women 36-55	Men 36-55	Women 56-65	Men 56-65	Adults	Retirees
**Lower Silesia mean**															
Vegetables	Root	-	-	-	-	-	-	-	-	-	-	-	-	-	-
	Leaf	**0.35**	**0.51**	**0.70**	3.56	3.37	1.12	6.26	6.17	5.72	6.19	5.64	5.87	**0.89**	6.31
	Fruit	-	-	-	-	-	-	-	-	-	-	-	-	-	-
	Inflorescence	-	-	-	-	-	-	-	-	-	-	-	-	-	-
	Legume	-	-	-	-	-	-	-	-	-	-	-	-	-	-
	Potatoes	-	-	-	-	-	-	-	-	-	-	-	-	-	-
Fruits	Berry	19.02	32.97	38.04	66.75	63.59	56.66	141.96	213.58	140.44	186.37	129.82	169.18	115.38	186.58
	Pome	-	-	-	-	-	-	-	-	-	-	-	-	-	-
	Stone	100.27	173.91	200.53	352.15	335.48	298.91	748.91	1126.72	740.89	983.18	684.87	892.50	607.64	1094.16
Cereals	Wheat	-	-	-	-	-	-	-	-	-	-	-	-	-	-
	Rye	-	-	-	-	-	-	-	-	-	-	-	-	-	-
	Barley	-	-	-	-	-	-	-	-	-	-	-	-	-	-
	Oat	-	-	-	-	-	-	-	-	-	-	-	-	-	-
**Lower Silesia P95**															
		Toddlers	Pre-schoolers	School-aged	Girls	Boys	Adolescents	Women 18-35	Men 18-35	Women 36-55	Men 36-55	Women 56-65	Men 56-65	Adults	Retirees
Vegetables	Root	-	-	-	-	-	-	-	-	-	-	-	-	-	-
	Leaf	**0.28**	**0.41**	**0.56**	2.86	2.71	**0.90**	5.03	4.95	4.59	4.97	4.53	4.71	**0.72**	5.07
	Fruit	-	-	-	-	-	-	-	-	-	-	-	-	-	-
	Inflorescence	-	-	-	-	-	-	-	-	-	-	-	-	-	-
	Legume	-	-	-	-	-	-	-	-	-	-	-	-	-	-
	Potatoes	-	-	-	-	-	-	-	-	-	-	-	-	-	-
Fruits	Berry	12.84	22.26	25.69	45.08	42.95	38.27	95.87	144.24	94.84	125.86	87.67	114.25	77.92	126.00
	Pome	-	-	-	-	-	-	-	-	-	-	-	-	-	-
	Stone	60.16	104.35	120.32	211.29	201.29	179.35	449.34	676.03	444.53	589.91	410.92	535.50	364.58	656.49
Cer	Wheat	-	-	-	-	-	-	-	-	-	-	-	-	-	-
	Rye	-	-	-	-	-	-	-	-	-	-	-	-	-	-

	Barley	-	-	-	-	-	-	-	-	-	-	-	-	-	-
	Oat	-	-	-	-	-	-	-	-	-	-	-	-	-	-
**Lublin mean**															
		Toddlers	Pre-schoolers	School-aged	Girls	Boys	Adolescents	Women 18-35	Men 18-35	Women 36-55	Men 36-55	Women 56-65	Men 56-65	Adults	Retirees
Vegetables	Root	**0.22**	**0.33**	**0.45**	1.27	1.21	**0.72**	2.24	2.20	2.04	2.21	2.02	2.10	**0.58**	2.26
	Leaf	**0.79**	1.16	1.58	8.11	7.69	2.55	14.25	14.04	13.02	14.09	12.86	13.37	2.03	14.37
	Fruit	3.02	4.42	6.03	17.10	16.20	9.73	30.05	29.61	27.45	29.71	27.10	28.18	7.74	30.29
	Inflorescence	23.24	34.09	46.49	131.79	124.88	75.00	231.56	228.17	211.50	228.97	208.86	217.16	59.66	233.42
	Legume	-	-	-	-	-	-	-	-	-	-	-	-	-	-
	Potatoes	-	-	-	4.91	4.65	-	9.30	6.65	8.52	7.32	8.73	7.36	-	11.35
Fruits	Berry	5.98	10.36	11.95	20.97	19.98	17.80	44.60	67.11	44.13	58.56	40.79	53.16	36.25	58.62
	Pome	-	-	-	-	-	-	-	-	-	-	-	-	-	-
	Stone	-	-	-	-	-	-	-	-	-	-	-	-	-	-
Cereals	Wheat	1.97	3.00	3.94	-	-	5.16	10.70	7.88	11.62	9.09	12.67	10.31	7.66	8.71
	Rye	4.89	7.46	9.77	-	-	12.82	73.71	54.31	80.08	62.61	87.27	71.04	19.02	59.98
	Barley	2.62	8.10	5.24	-	-	13.92	14.88	10.96	16.16	12.64	17.61	14.34	20.59	12.11
	Oat	6.09	46.60	12.17	-	-	80.88	36.10	26.60	39.22	30.66	42.74	34.79	117.65	29.37
**Lublin P95**															
		Toddlers	Pre-schoolers	School-aged	Girls	Boys	Adolescents	Women 18-35	Men 18-35	Women 36-55	Men 36-55	Women 56-65	Men 56-65	Adults	Retirees
Vegetables	Root	0.15	0.22	0.30	0.84	0.80	0.48	1.48	1.45	1.35	1.46	1.33	1.38	0.38	1.49
	Leaf	0.26	0.38	0.52	2.66	2.52	0.84	4.68	4.61	4.27	4.62	4.22	4.39	0.67	4.71
	Fruit	0.54	0.80	1.09	3.09	2.93	1.76	5.43	5.35	4.96	5.37	4.90	5.09	1.40	5.47
	Inflorescence	23.24	34.09	46.49	131.79	124.88	75.00	231.56	228.17	211.50	228.97	208.86	217.16	59.66	233.42
	Legume	-	-	-	-	-	-	-	-	-	-	-	-	-	-
	Potatoes	-	-	-	3.71	3.52	-	7.03	5.03	6.44	5.54	6.60	5.57	-	8.59
Fruits	Berry	1.97	3.42	3.95	6.93	6.60	5.88	14.74	22.18	14.58	19.35	13.48	17.57	11.98	19.37
	Pome	-	-	-	-	-	-	-	-	-	-	-	-	-	-
	Stone	-	-	-	-	-	-	-	-	-	-	-	-	-	-
Cereal	Wheat	1.25	1.91	2.50	-	-	3.28	6.80	5.01	7.39	5.78	8.06	6.56	4.87	5.54
	Rye	2.38	3.64	4.77	-	-	6.25	35.96	26.49	39.06	30.54	42.57	34.65	9.28	29.26
	Barley	1.77	5.47	3.54	-	-	9.41	10.05	7.41	10.92	8.54	11.90	9.69	13.91	8.18

	Oat	4.27	32.74	8.55	-	-	56.82	25.36	18.68	27.55	21.54	30.02	24.44	82.64	20.63
**Lesser Poland mean**															
		Toddlers	Pre-schoolers	School-aged	Girls	Boys	Adolescents	Women 18-35	Men 18-35	Women 36-55	Men 36-55	Women 56-65	Men 56-65	Adults	Retirees
Vegetables	Root	5.13	7.53	10.27	29.11	27.59	16.57	51.15	50.40	46.72	50.58	46.14	47.97	13.18	51.56
	Leaf	**0.37**	**0.54**	**0.73**	3.75	3.55	1.18	6.58	6.49	6.01	6.51	5.94	6.18	**0.94**	6.64
	Fruit	1.29	1.89	2.57	7.30	6.91	4.15	12.82	12.63	11.71	12.68	11.56	12.02	3.30	12.92
	Inflorescence	-	-	-	-	-	-	-	-	-	-	-	-	-	-
	Legume	19.97	30.44	39.95	680.74	645.00	53.27	902.38	1065.31	952.51	1127.97	971.31	1121.70	46.61	599.73
	Potatoes	-	-	-	3.33	3.15	-	6.30	4.51	5.77	4.96	5.91	4.98	-	7.69
Fruits	Berry	3.56	6.16	7.11	12.48	11.89	10.59	26.54	39.93	26.26	34.84	24.27	31.63	21.57	34.88
	Pome	-	-	-	-	-	-	-	-	-	-	-	-	-	-
	Stone	**0.55**	**0.95**	1.09	1.92	1.83	1.63	4.09	6.15	4.04	5.36	3.74	4.87	3.31	5.97
Cereals	Wheat	-	-	-	-	-	-	-	-	-	-	-	-	-	-
	Rye	-	-	-	-	-	-	-	-	-	-	-	-	-	-
	Barley	-	-	-	-	-	-	-	-	-	-	-	-	-	-
	Oat	-	-	-	-	-	-	-	-	-	-	-	-	-	-
**Lesser Poland P95**															
		Toddlers	Pre-schoolers	School-aged	Girls	Boys	Adolescents	Women 18-35	Men 18-35	Women 36-55	Men 36-55	Women 56-65	Men 56-65	Adults	Retirees
Vegetables	Root	1.21	1.78	2.42	6.86	6.50	3.91	12.06	11.88	11.02	11.93	10.88	11.31	3.11	12.16
	Leaf	**0.07**	**0.11**	**0.15**	**0.77**	**0.73**	**0.24**	1.35	1.33	1.23	1.33	1.22	1.26	**0.19**	1.36
	Fruit	**0.36**	**0.54**	**0.73**	2.07	1.96	1.18	3.63	3.58	3.32	3.59	3.28	3.41	**0.94**	3.66
	Inflorescence	-	-	-	-	-	-	-	-	-	-	-	-	-	-
	Legume	-	-	-	-	-	-	-	-	-	-	-	-	-	-
	Potatoes	-	-	-	1.48	1.40	-	2.80	2.00	2.58	2.20	2.63	5.57	-	8.59
Fruits	Berry	1.24	2.16	2.49	4.37	4.16	3.71	9.29	13.97	9.19	12.19	8.49	11.07	7.55	12.20
	Pome	-	-	-	-	-	-	-	-	-	-	-	-	-	-
	Stone	**0.18**	**0.31**	**0.36**	**0.62**	**0.59**	**0.53**	1.33	2.00	1.31	1.74	1.21	1.58	1.08	1.94
Cereals	Wheat	-	-	-	-	-	-	-	-	-	-	-	-	-	-
	Rye	-	-	-	-	-	-	-	-	-	-	-	-	-	-
	Barley	-	-	-	-	-	-	-	-	-	-	-	-	-	-
	Oat	-	-	-	-	-	-	-	-	-	-	-	-	-	-

Northern Poland mean															
		Toddlers	Pre-schoolers	School-aged	Girls	Boys	Adolescents	Women 18-35	Men 18-35	Women 36-55	Men 36-55	Women 56-65	Men 56-65	Adults	Retirees
Vegetables	Root	2.08	3.05	4.17	11.81	11.19	6.72	20.75	20.45	18.95	20.52	18.71	19.45	5.35	20.92
	Leaf	2.27	3.33	4.55	23.29	22.07	7.33	40.92	40.32	37.37	40.46	36.91	38.37	5.83	41.25
	Fruit	1.82	2.67	3.64	10.32	9.78	5.88	18.14	17.87	16.57	17.94	16.36	17.01	4.67	18.28
	Inflorescence	7.48	10.96	14.95	42.39	40.16	24.12	74.48	73.39	68.03	73.64	67.18	69.85	19.19	75.08
	Legume	7.53	11.47	15.05	256.46	242.99	20.07	339.96	401.34	358.84	424.95	365.93	422.59	17.56	225.98
	Potatoes	-	-	-	2.58	2.44	-	4.88	3.49	4.47	3.84	4.58	3.86	-	5.96
Fruits	Berry	-	-	-	-	-	-	-	-	-	-	-	-	-	-
	Pome	-	-	-	-	-	-	-	-	-	-	-	-	-	-
	Stone	-	-	-	-	-	-	-	-	-	-	-	-	-	-
Cereals	Wheat	1.94	2.95	3.88	-	-	5.08	10.53	7.76	11.44	8.94	12.47	10.15	7.54	8.57
	Rye	-	-	-	-	-	-	-	-	-	-	-	-	-	-
	Barley	4.52	13.96	9.03	-	-	24.01	25.65	18.90	27.86	21.79	30.37	24.72	35.50	20.87
	Oat	-	-	-	-	-	-	-	-	-	-	-	-	-	-

Northern Poland P95															
		Toddlers	Pre-schoolers	School-aged	Girls	Boys	Adolescents	Women 18-35	Men 18-35	Women 36-55	Men 36-55	Women 56-65	Men 56-65	Adults	Retirees
Vegetables	Root	1.39	2.04	2.78	7.89	7.48	4.49	13.87	13.67	12.67	13.71	12.51	13.01	3.57	13.98
	Leaf	1.12	1.65	2.25	11.52	10.92	3.63	20.25	19.95	18.49	20.02	18.26	18.99	2.89	20.41
	Fruit	1.53	2.24	3.05	8.65	8.19	4.92	15.19	14.97	13.88	15.02	13.70	14.25	3.91	15.31
	Inflorescence	6.18	9.06	12.35	35.02	33.18	19.93	61.53	60.62	56.20	60.84	55.49	57.70	15.85	62.02
	Legume	4.63	7.05	9.26	157.71	149.43	12.34	209.06	246.81	220.68	261.33	225.03	259.88	10.80	138.95
	Potatoes	-	-	-	2.58	2.44	-	4.88	3.49	4.47	3.84	4.58	3.86	-	5.96
Fruits	Berry	-	-	-	-	-	-	-	-	-	-	-	-	-	-
	Pome	-	-	-	-	-	-	-	-	-	-	-	-	-	-
	Stone	-	-	-	-	-	-	-	-	-	-	-	-	-	-
Cereals	Wheat	1.40	2.14	2.81	-	-	3.68	7.63	5.62	8.28	6.48	9.03	7.35	5.46	6.20
	Rye	-	-	-	-	-	-	-	-	-	-	-	-	-	-
	Barley	3.39	10.46	6.77	-	-	17.99	19.22	14.16	20.88	16.33	22.76	18.52	26.60	15.64
	Oat	-	-	-	-	-	-	-	-	-	-	-	-	-	-

Opole mean

		Toddlers	Pre-schoolers	School-aged	Girls	Boys	Adolescents	Women 18-35	Men 18-35	Women 36-55	Men 36-55	Women 56-65	Men 56-65	Adults	Retirees
Vegetables	Root	23.76	34.85	47.53	134.74	127.67	76.68	236.74	233.27	216.23	234.09	213.53	222.02	60.99	238.64
	Leaf	1.28	1.88	2.56	13.13	12.44	4.13	23.07	22.73	21.07	22.81	20.81	21.63	3.29	23.25
	Fruit	19.42	28.48	38.84	110.11	104.32	62.66	193.45	190.62	176.70	191.29	174.49	181.42	49.84	195.00
	Inflorescence	-	-	-	-	-	-	-	-	-	-	-	-	-	-
	Legume	4.51	6.88	9.03	153.79	145.72	12.03	203.86	240.67	215.19	254.83	219.44	253.41	10.53	135.49
	Potatoes	-	-	-	9.78	9.26	-	18.51	13.25	16.96	14.58	17.38	14.65	-	22.61
Fruits	Berry	2.22	3.85	4.44	7.80	7.43	6.62	16.58	24.95	16.41	21.77	15.17	19.76	13.48	21.80
	Pome	33.62	58.30	67.25	118.04	112.46	100.20	251.04	377.69	248.35	329.57	229.58	299.18	203.88	285.17
	Stone	3.31	5.74	6.62	11.62	11.07	9.86	24.71	37.18	24.45	32.44	22.60	29.45	20.05	36.10
Cereals	Wheat	-	-	-	-	-	-	-	-	-	-	-	-	-	-
	Rye	-	-	-	-	-	-	-	-	-	-	-	-	-	-
	Barley	-	-	-	-	-	-	-	-	-	-	-	-	-	-
	Oat	-	-	-	-	-	-	-	-	-	-	-	-	-	-

Opole P95

		Toddlers	Pre-schoolers	School-aged	Girls	Boys	Adolescents	Women 18-35	Men 18-35	Women 36-55	Men 36-55	Women 56-65	Men 56-65	Adults	Retirees
Vegetables	Root	5.30	7.77	10.59	30.03	28.46	17.09	52.77	51.99	48.20	52.18	47.59	49.49	13.59	53.19
	Leaf	1.29	1.90	2.59	13.26	12.56	4.17	23.29	22.95	21.27	23.03	21.01	21.84	3.32	23.48
	Fruit	4.88	7.16	9.76	27.66	26.21	15.74	48.60	47.89	44.39	48.06	43.84	45.58	12.52	48.99
	Inflorescence	-	-	-	-	-	-	-	-	-	-	-	-	-	-
	Legume	1.19	1.81	2.38	40.56	38.43	3.17	53.77	63.47	56.75	67.21	57.87	66.83	2.78	35.73
	Potatoes	-	-	-	3.60	3.41	-	6.82	4.88	6.25	5.37	6.40	5.40	-	8.33
Fruits	Berry	0.47	0.81	0.94	1.64	1.56	1.39	3.49	5.25	3.45	4.58	3.19	4.16	2.84	4.59
	Pome	10.00	17.34	20.00	35.11	33.45	29.80	74.67	112.33	73.87	98.02	68.28	88.98	60.64	84.82
	Stone	1.60	2.77	3.20	5.62	5.35	4.77	11.95	17.98	11.82	15.69	10.93	14.24	9.70	17.46
Cereals	Wheat	-	-	-	-	-	-	-	-	-	-	-	-	-	-
	Rye	-	-	-	-	-	-	-	-	-	-	-	-	-	-
	Barley	-	-	-	-	-	-	-	-	-	-	-	-	-	-
	Oat	-	-	-	-	-	-	-	-	-	-	-	-	-	-

Silesia mean

		Toddlers	Pre-schoolers	School-aged	Girls	Boys	Adolescents	Women 18-35	Men 18-35	Women 36-55	Men 36-55	Women 56-65	Men 56-65	Adults	Retirees
Vegetables	Root	**0.12**	**0.17**	**0.24**	**0.68**	**0.64**	**0.38**	1.19	1.17	1.08	1.17	1.07	1.11	**0.31**	1.20
	Leaf	1.82	2.68	3.65	18.69	17.71	5.89	32.84	32.36	29.99	32.47	29.62	30.80	4.68	33.10
	Fruit	-	-	-	-	-	-	-	-	-	-	-	-	-	-
	Inflorescence	13.46	19.74	26.91	76.30	72.30	43.42	134.06	132.10	122.45	132.56	120.92	125.73	34.54	135.14
	Legume	-	-	-	-	-	-	-	-	-	-	-	-	-	-
	Potatoes	-	-	-	1.50	1.42	-	2.84	2.03	2.60	2.24	2.67	2.25	-	3.47
Fruits	Berry	1.20	2.08	2.40	4.21	4.02	3.58	8.96	13.49	8.87	11.77	8.20	10.68	7.29	3.52
	Pome	**0.62**	1.07	1.23	2.16	2.06	1.83	4.59	6.91	4.54	6.03	4.20	5.47	3.73	5.22
	Stone	7.92	13.74	15.84	27.82	26.50	23.61	59.15	89.00	58.52	77.66	54.10	70.50	48.00	86.42
Cereals	Wheat	-	-	-	-	-	-	-	-	-	-	-	-	-	-
	Rye	-	-	-	-	-	-	-	-	-	-	-	-	-	-
	Barley	-	-	-	-	-	-	-	-	-	-	-	-	-	-
	Oat	-	-	-	-	-	-	-	-	-	-	-	-	-	-
**Silesia P95**															
		Toddlers	Pre-schoolers	School-aged	Girls	Boys	Adolescents	Women 18-35	Men 18-35	Women 36-55	Men 36-55	Women 56-65	Men 56-65	Adults	Retirees
Vegetables	Root	**0.04**	**0.06**	**0.08**	**0.24**	**0.23**	**0.14**	**0.42**	**0.41**	**0.38**	**0.41**	**0.38**	**0.39**	**0.11**	**0.42**
	Leaf	**0.61**	**0.89**	1.21	6.22	5.89	1.96	10.92	10.76	9.98	10.80	9.85	10.24	1.56	11.01
	Fruit	-	-	-	-	-	-	-	-	-	-	-	-	-	-
	Inflorescence	13.46	19.74	26.91	76.30	72.30	43.42	134.06	132.10	122.45	132.56	120.92	125.73	34.54	135.14
	Legume	-	-	-	-	-	-	-	-	-	-	-	-	-	-
	Potatoes	-	-	-	**0.59**	**0.56**	-	1.11	**0.80**	1.02	**0.88**	1.04	**0.88**	-	1.36
Fruits	Berry	**0.38**	**0.66**	**0.76**	1.34	1.27	1.13	2.84	4.28	2.81	3.73	2.60	3.39	2.31	3.52
	Pome	**0.34**	**0.59**	**0.68**	1.20	1.14	1.02	2.56	3.84	2.53	3.35	2.34	3.05	2.08	2.90
	Stone	2.36	4.10	4.73	8.30	7.91	7.05	17.65	26.56	17.46	23.17	16.14	21.04	14.32	25.79
Cereals	Wheat	-	-	-	-	-	-	-	-	-	-	-	-	-	-
	Rye	-	-	-	-	-	-	-	-	-	-	-	-	-	-
	Barley	-	-	-	-	-	-	-	-	-	-	-	-	-	-
	Oat	-	-	-	-	-	-	-	-	-	-	-	-	-	-

Lower Silesia

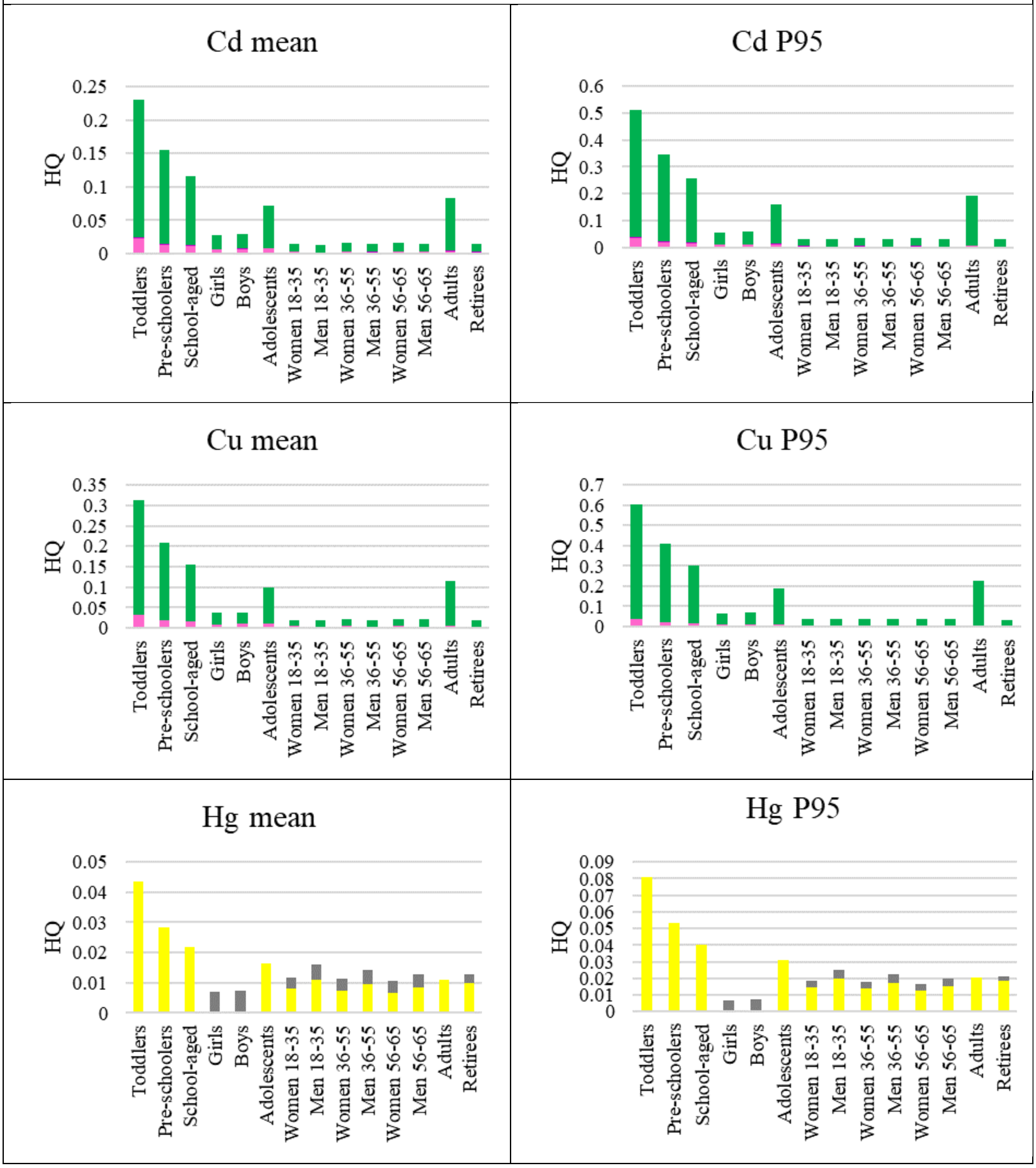

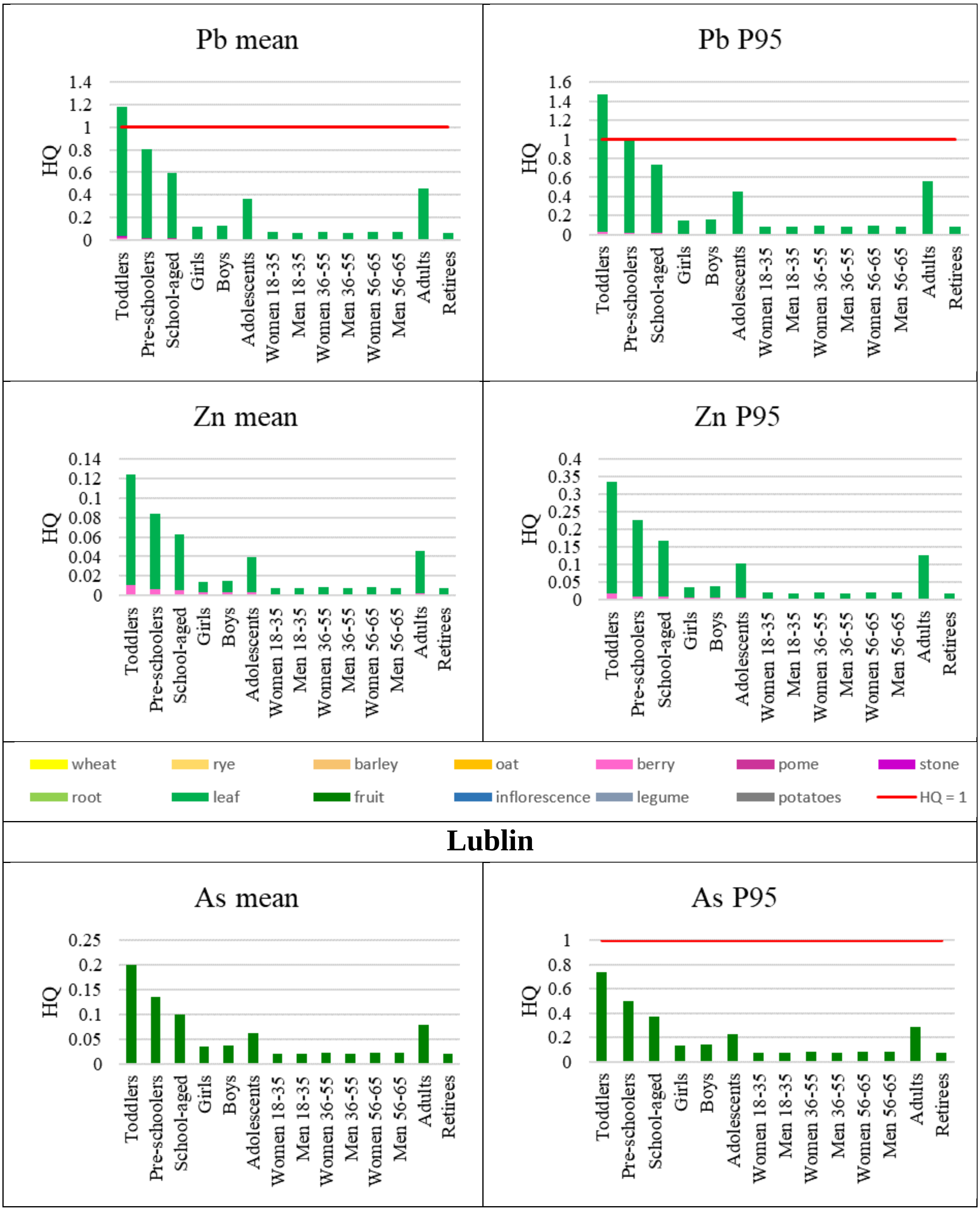
Pb mean
Pb P95
Zn mean
Zn P95
HQ
Toddlers
Pre-schoolers
School-aged
Girls
Boys
Adolescents
Women 18-35
Men 18-35
Women 36-55
Men 36-55
Women 56-65
Men 56-65
Adults
Retirees
wheat
rye
barley
oat
berry
pome
stone
root
leaf
fruit
inflorescence
legume
potatoes
HQ = 1
Lublin
As mean
As P95

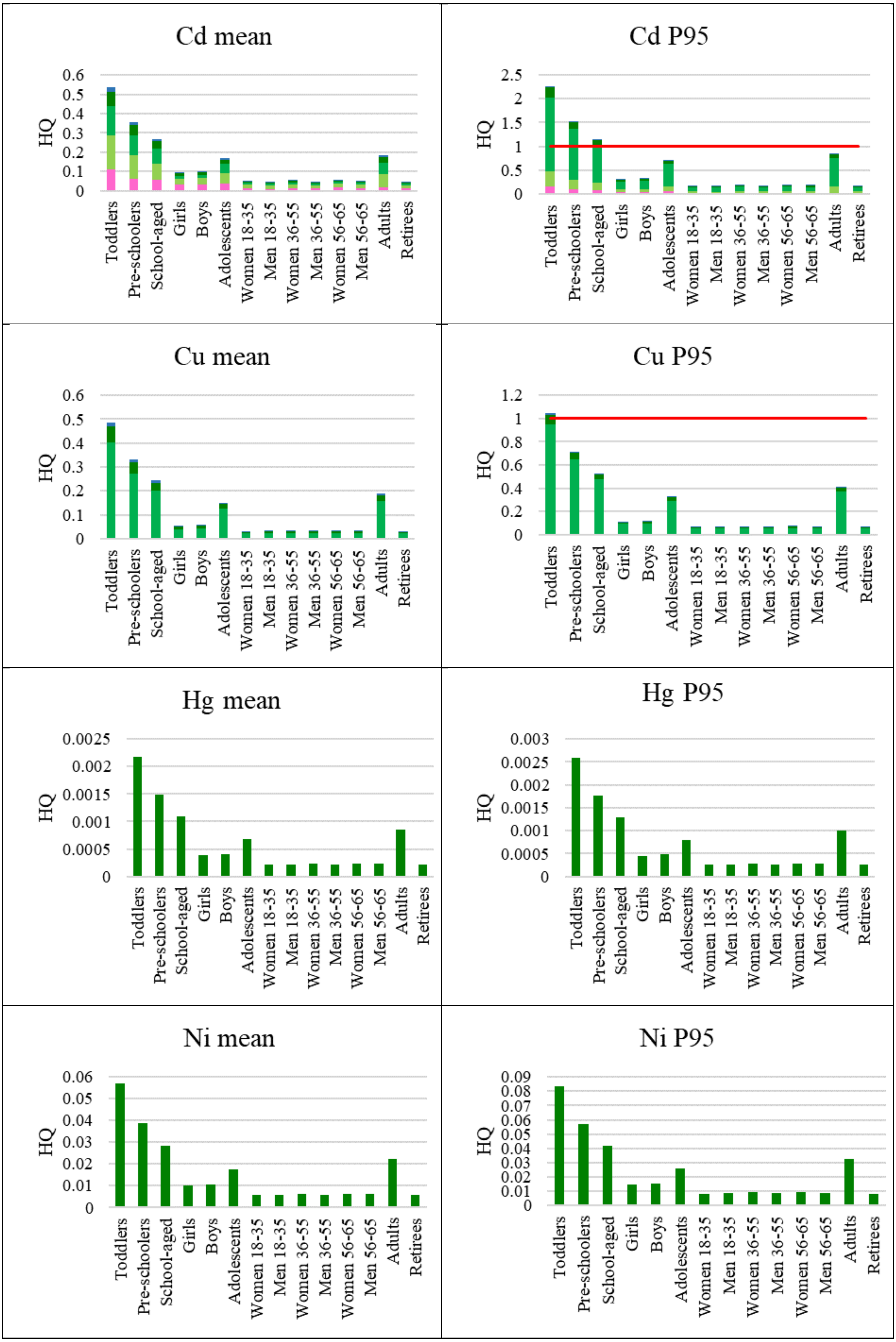
Cd mean
HQ
0.6
0.5
0.4
0.3
0.2
0.1
0
Toddlers
Pre-schoolers
School-aged
Girls
Boys
Adolescents
Women 18-35
Men 18-35
Women 36-55
Men 36-55
Women 56-65
Men 56-65
Adults
Retirees
Cd P95
HQ
2.5
2
1.5
1
0.5
0
Toddlers
Pre-schoolers
School-aged
Girls
Boys
Adolescents
Women 18-35
Men 18-35
Women 36-55
Men 36-55
Women 56-65
Men 56-65
Adults
Retirees
Cu mean
HQ
0.6
0.5
0.4
0.3
0.2
0.1
0
Toddlers
Pre-schoolers
School-aged
Girls
Boys
Adolescents
Women 18-35
Men 18-35
Women 36-55
Men 36-55
Women 56-65
Men 56-65
Adults
Retirees
Cu P95
HQ
1.2
1
0.8
0.6
0.4
0.2
0
Toddlers
Pre-schoolers
School-aged
Girls
Boys
Adolescents
Women 18-35
Men 18-35
Women 36-55
Men 36-55
Women 56-65
Men 56-65
Adults
Retirees
Hg mean
HQ
0.0025
0.002
0.0015
0.001
0.0005
0
Toddlers
Pre-schoolers
School-aged
Girls
Boys
Adolescents
Women 18-35
Men 18-35
Women 36-55
Men 36-55
Women 56-65
Men 56-65
Adults
Retirees
Hg P95
HQ
0.003
0.0025
0.002
0.0015
0.001
0.0005
0
Toddlers
Pre-schoolers
School-aged
Girls
Boys
Adolescents
Women 18-35
Men 18-35
Women 36-55
Men 36-55
Women 56-65
Men 56-65
Adults
Retirees
Ni mean
HQ
0.06
0.05
0.04
0.03
0.02
0.01
0
Toddlers
Pre-schoolers
School-aged
Girls
Boys
Adolescents
Women 18-35
Men 18-35
Women 36-55
Men 36-55
Women 56-65
Men 56-65
Adults
Retirees
Ni P95
HQ
0.09
0.08
0.07
0.06
0.05
0.04
0.03
0.02
0.01
0
Toddlers
Pre-schoolers
School-aged
Girls
Boys
Adolescents
Women 18-35
Men 18-35
Women 36-55
Men 36-55
Women 56-65
Men 56-65
Adults
Retirees

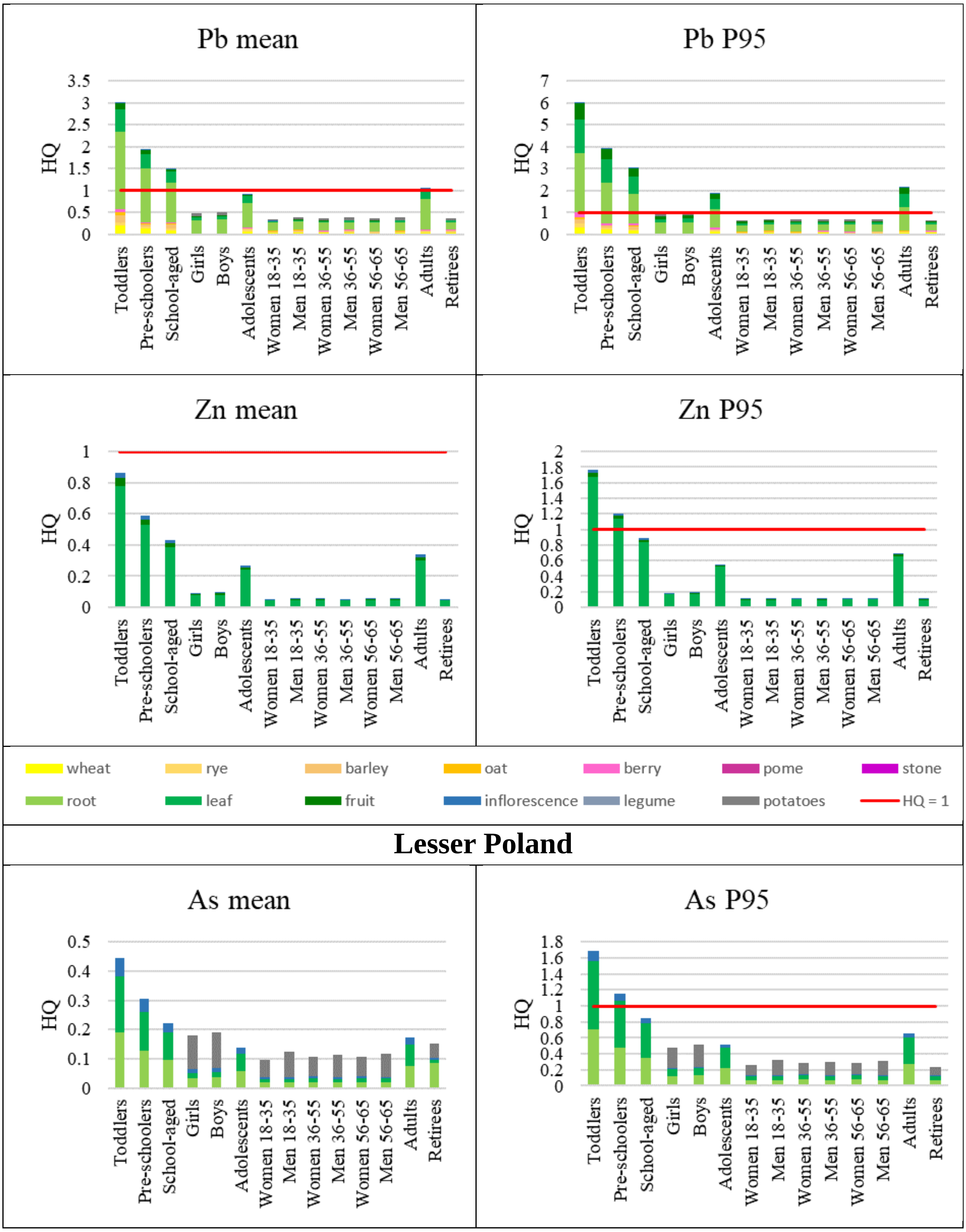
Pb mean
Pb P95
Zn mean
Zn P95
As mean
As P95
HQ
Toddlers
Pre-schoolers
School-aged
Girls
Boys
Adolescents
Women 18-35
Men 18-35
Women 36-55
Men 36-55
Women 56-65
Men 56-65
Adults
Retirees
wheat
rye
barley
oat
berry
pome
stone
root
leaf
fruit
inflorescence
legume
potatoes
HQ = 1
Lesser Poland

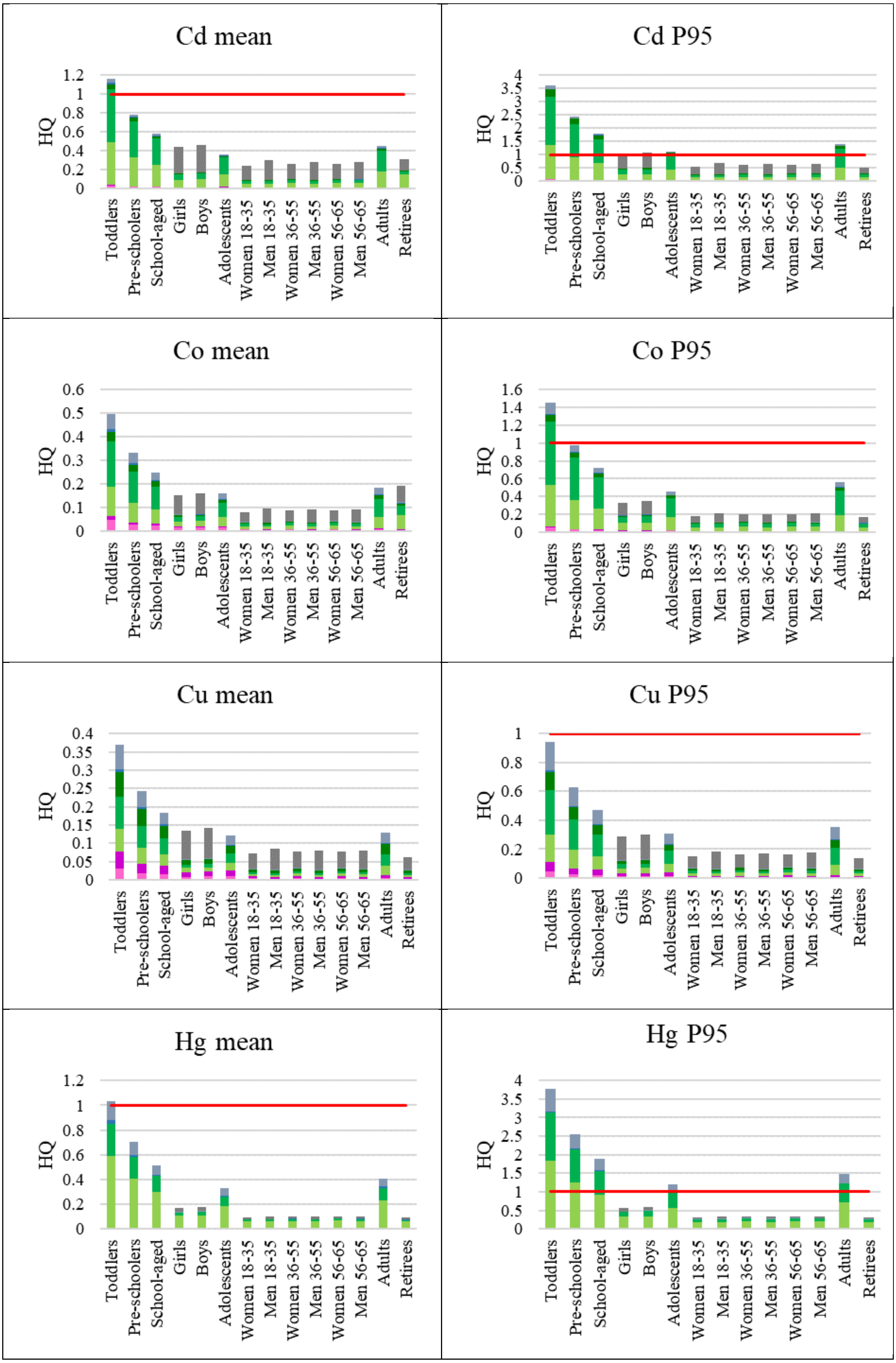
Cd mean
Cd P95
Co mean
Co P95
Cu mean
Cu P95
Hg mean
Hg P95
HQ
Toddlers
Pre-schoolers
School-aged
Girls
Boys
Adolescents
Women 18-35
Men 18-35
Women 36-55
Men 36-55
Women 56-65
Men 56-65
Adults
Retirees

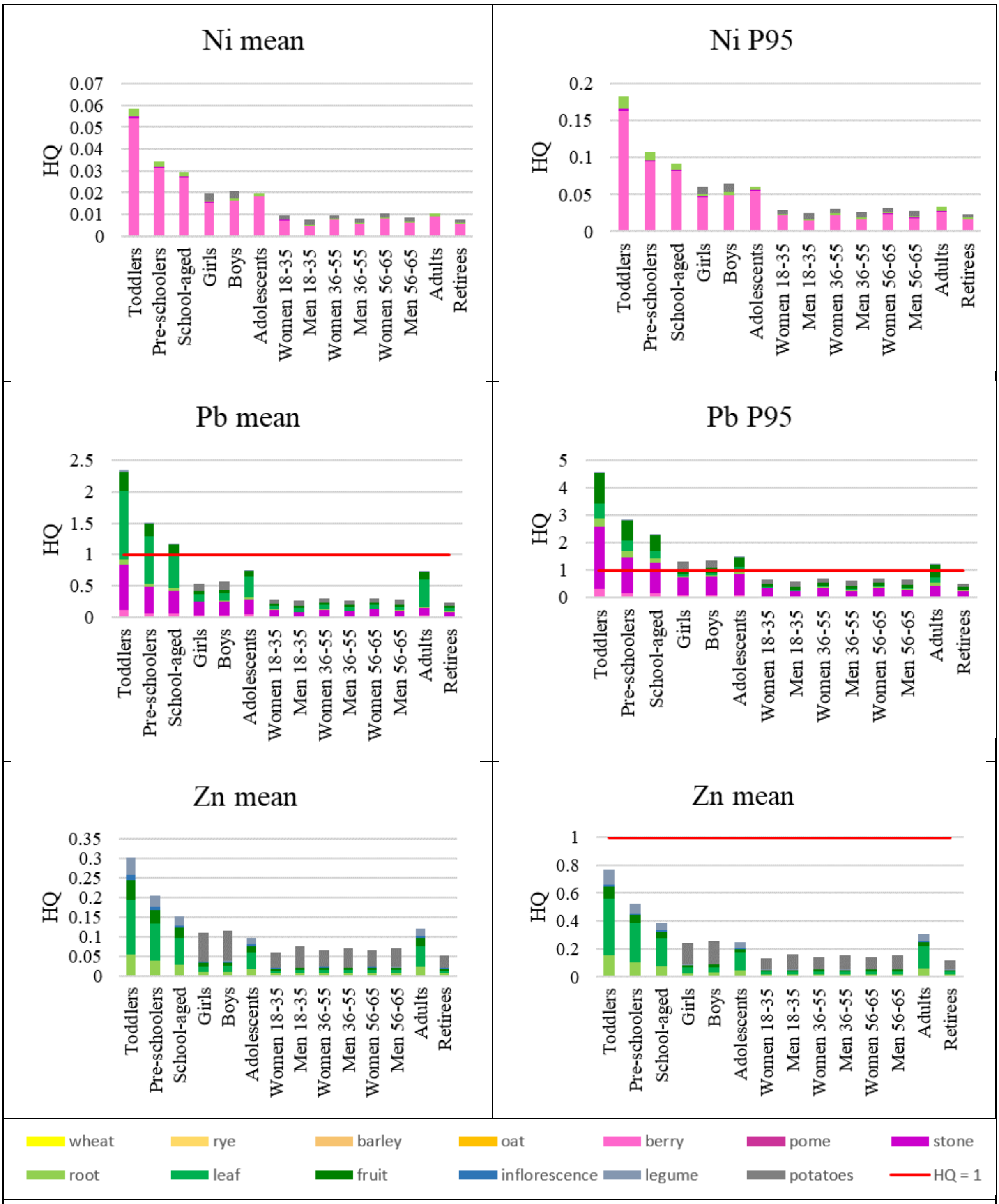

Northern Poland

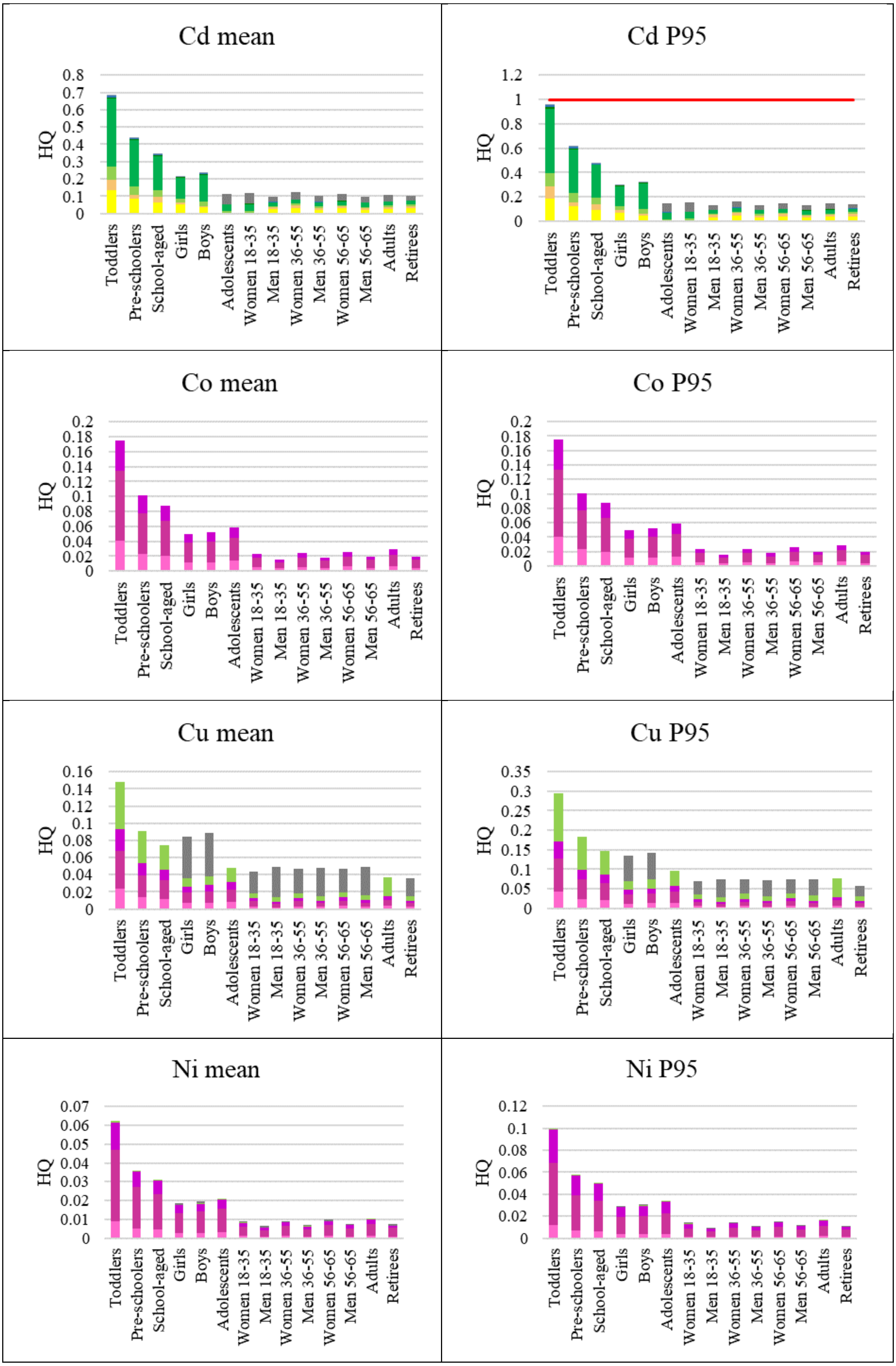
Cd mean
HQ
0 0.1 0.2 0.3 0.4 0.5 0.6 0.7 0.8
Toddlers Pre-schoolers School-aged Girls Boys Adolescents Women 18-35 Men 18-35 Women 36-55 Men 36-55 Women 56-65 Men 56-65 Adults Retirees
Cd P95
HQ
0 0.2 0.4 0.6 0.8 1 1.2
Toddlers Pre-schoolers School-aged Girls Boys Adolescents Women 18-35 Men 18-35 Women 36-55 Men 36-55 Women 56-65 Men 56-65 Adults Retirees
Co mean
HQ
0 0.02 0.04 0.06 0.08 0.1 0.12 0.14 0.16 0.18 0.2
Toddlers Pre-schoolers School-aged Girls Boys Adolescents Women 18-35 Men 18-35 Women 36-55 Men 36-55 Women 56-65 Men 56-65 Adults Retirees
Co P95
HQ
0 0.02 0.04 0.06 0.08 0.1 0.12 0.14 0.16 0.18 0.2
Toddlers Pre-schoolers School-aged Girls Boys Adolescents Women 18-35 Men 18-35 Women 36-55 Men 36-55 Women 56-65 Men 56-65 Adults Retirees
Cu mean
HQ
0 0.02 0.04 0.06 0.08 0.1 0.12 0.14 0.16
Toddlers Pre-schoolers School-aged Girls Boys Adolescents Women 18-35 Men 18-35 Women 36-55 Men 36-55 Women 56-65 Men 56-65 Adults Retirees
Cu P95
HQ
0 0.05 0.1 0.15 0.2 0.25 0.3 0.35
Toddlers Pre-schoolers School-aged Girls Boys Adolescents Women 18-35 Men 18-35 Women 36-55 Men 36-55 Women 56-65 Men 56-65 Adults Retirees
Ni mean
HQ
0 0.01 0.02 0.03 0.04 0.05 0.06 0.07
Toddlers Pre-schoolers School-aged Girls Boys Adolescents Women 18-35 Men 18-35 Women 36-55 Men 36-55 Women 56-65 Men 56-65 Adults Retirees
Ni P95
HQ
0 0.02 0.04 0.06 0.08 0.1 0.12
Toddlers Pre-schoolers School-aged Girls Boys Adolescents Women 18-35 Men 18-35 Women 36-55 Men 36-55 Women 56-65 Men 56-65 Adults Retirees

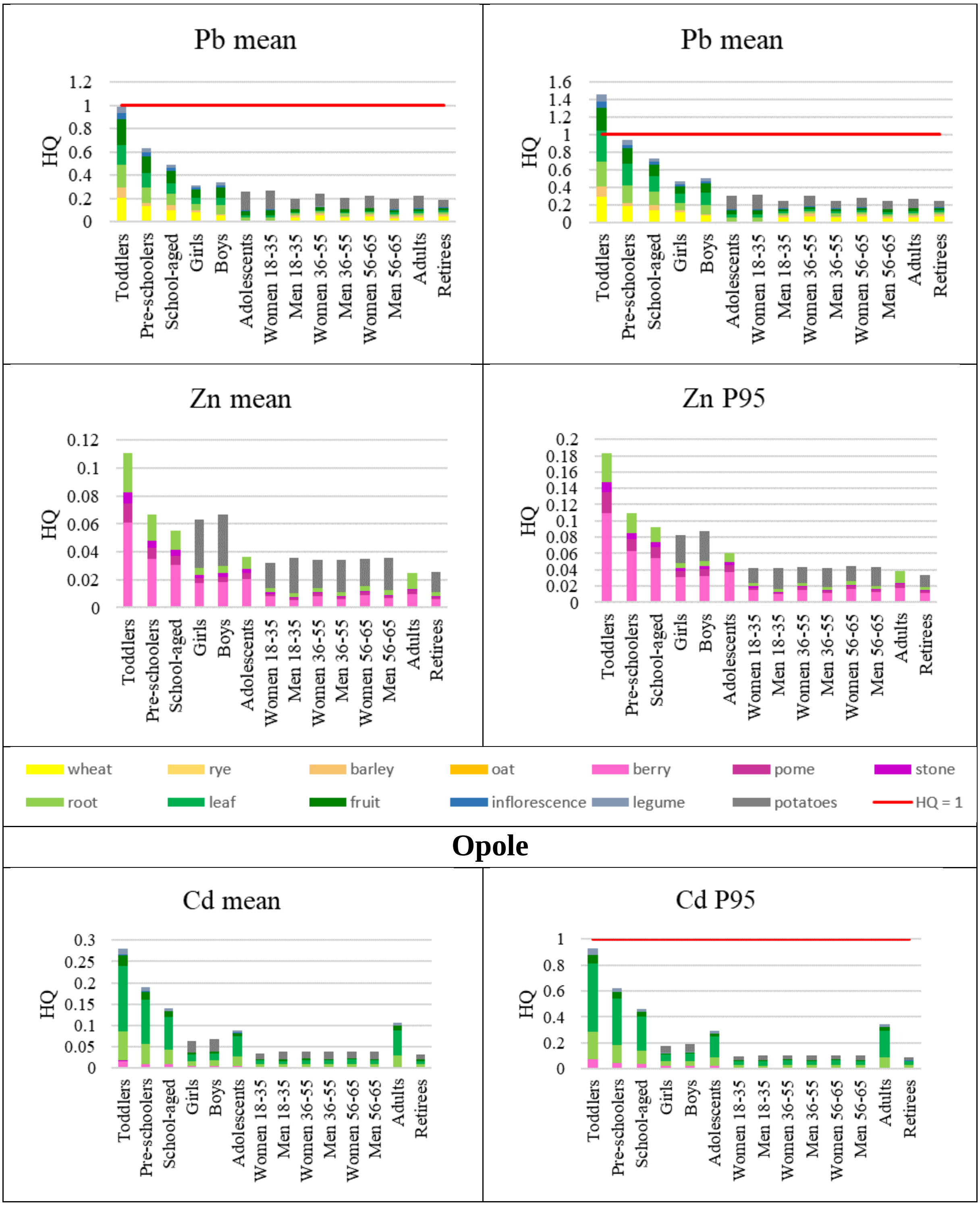
Pb mean
HQ
1.2
1
0.8
0.6
0.4
0.2
0
Toddlers
Pre-schoolers
School-aged
Girls
Boys
Adolescents
Women 18-35
Men 18-35
Women 36-55
Men 36-55
Women 56-65
Men 56-65
Adults
Retirees
Pb mean
1.6
1.4
1.2
Zn mean
0.12
0.1
0.08
0.06
0.04
0.02
Zn P95
0.2
0.18
0.16
0.14
wheat
rye
barley
oat
berry
pome
stone
root
leaf
fruit
inflorescence
legume
potatoes
HQ = 1
Opole
Cd mean
0.3
0.25
0.15
0.05
Cd P95

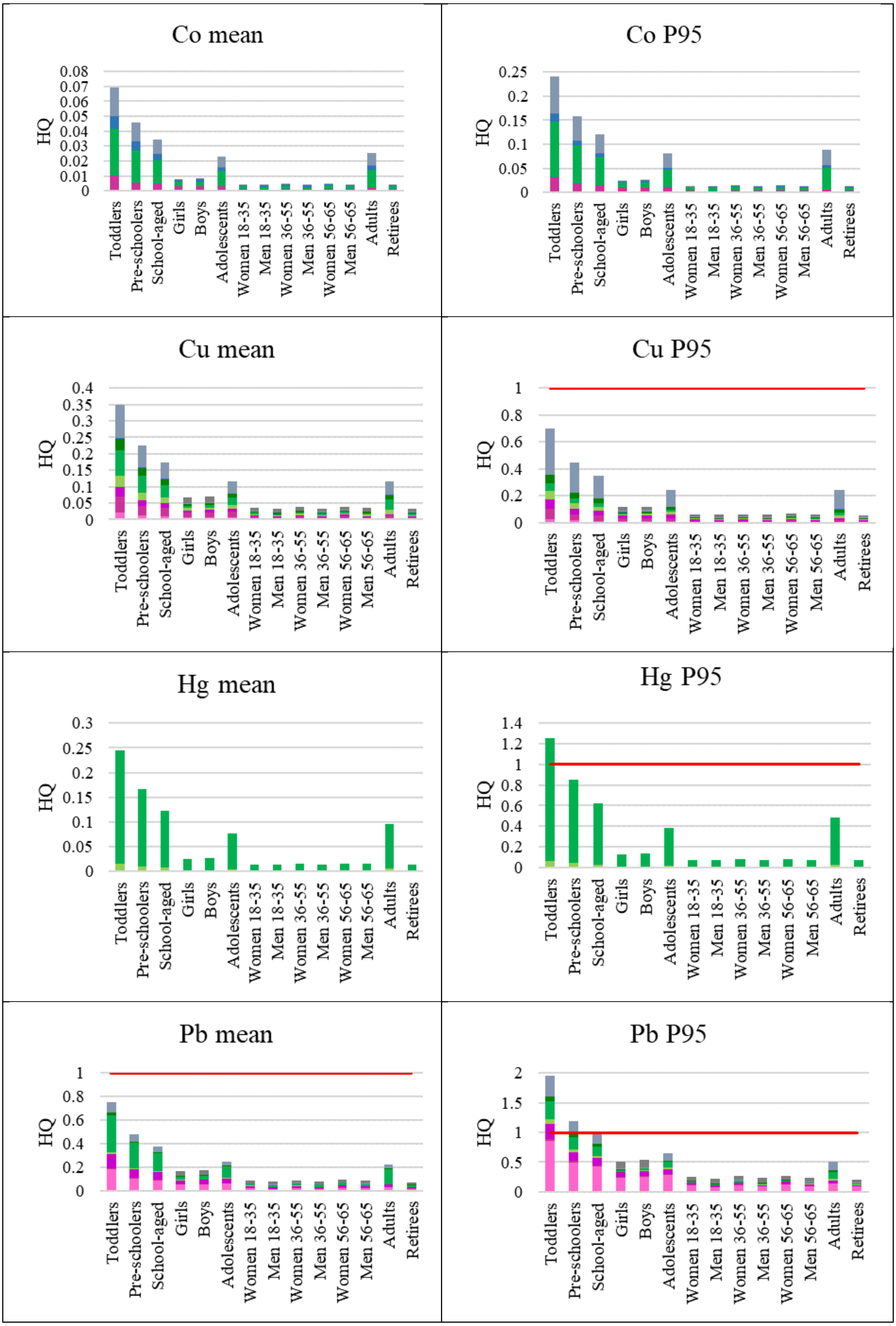
Co mean
HQ
0.08
0.07
0.06
0.05
0.04
0.03
0.02
0.01
0
Co P95
0.25
0.2
0.15
0.1
0.05
Cu mean
0.4
0.35
0.3
0.25
0.15
Cu P95
1
0.8
0.6
0.4
Hg mean
Hg P95
1.4
1.2
Pb mean
Pb P95
2
1.5
0.5
Toddlers
Pre-schoolers
School-aged
Girls
Boys
Adolescents
Women 18-35
Men 18-35
Women 36-55
Men 36-55
Women 56-65
Men 56-65
Adults
Retirees

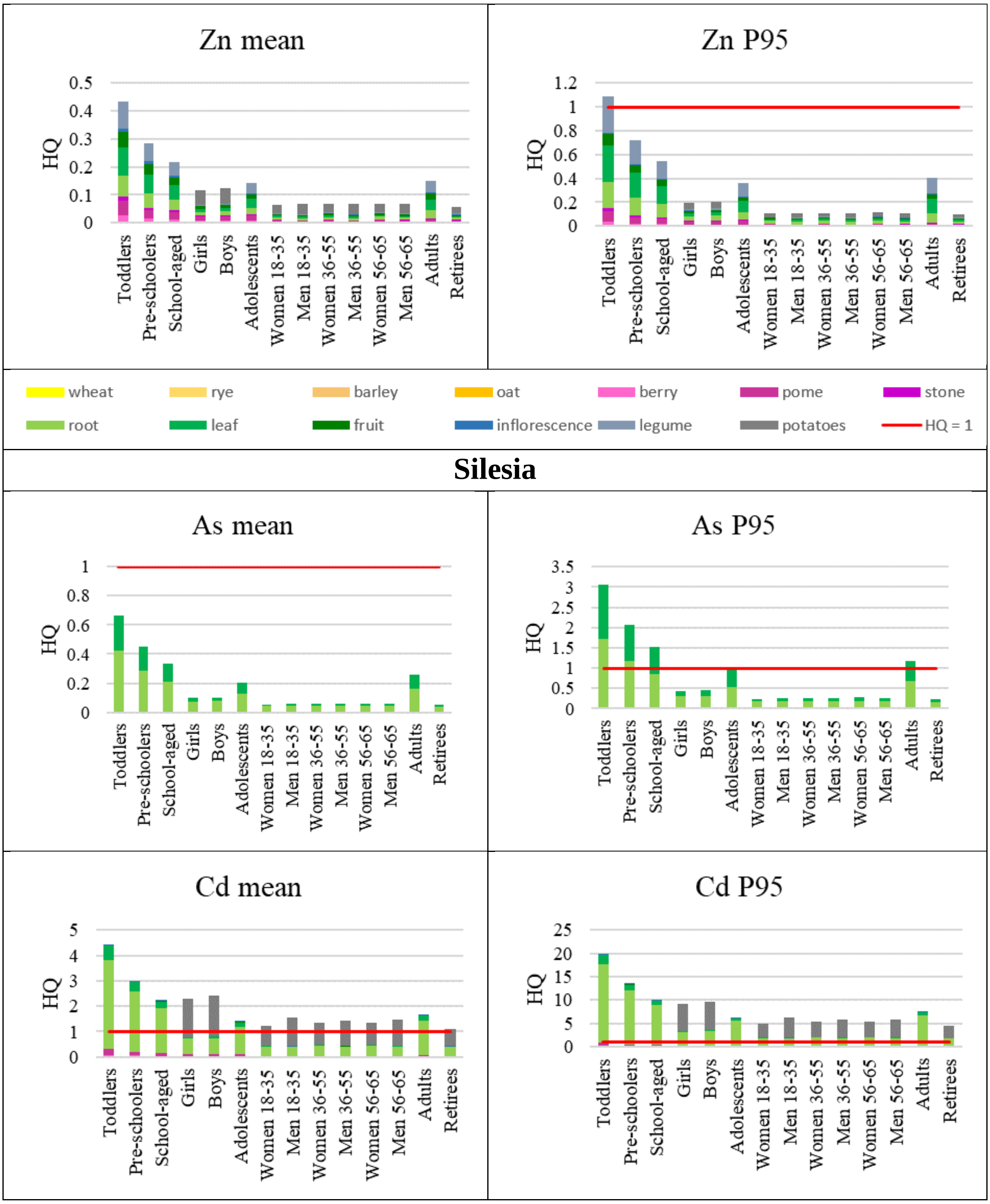
Zn mean
HQ
0.5
0.4
0.3
0.2
0.1
0
Toddlers
Pre-schoolers
School-aged
Girls
Boys
Adolescents
Women 18-35
Men 18-35
Women 36-55
Men 36-55
Women 56-65
Men 56-65
Adults
Retirees
Zn P95
HQ
1.2
1
0.8
0.6
0.4
0.2
0
wheat
rye
barley
oat
berry
pome
stone
root
leaf
fruit
inflorescence
legume
potatoes
HQ = 1
Silesia
As mean
HQ
1
0.8
0.6
0.4
0.2
0
As P95
HQ
3.5
3
2.5
2
1.5
1
0.5
0
Cd mean
HQ
5
4
3
2
1
0
Cd P95
HQ
25
20
15
10
5
0

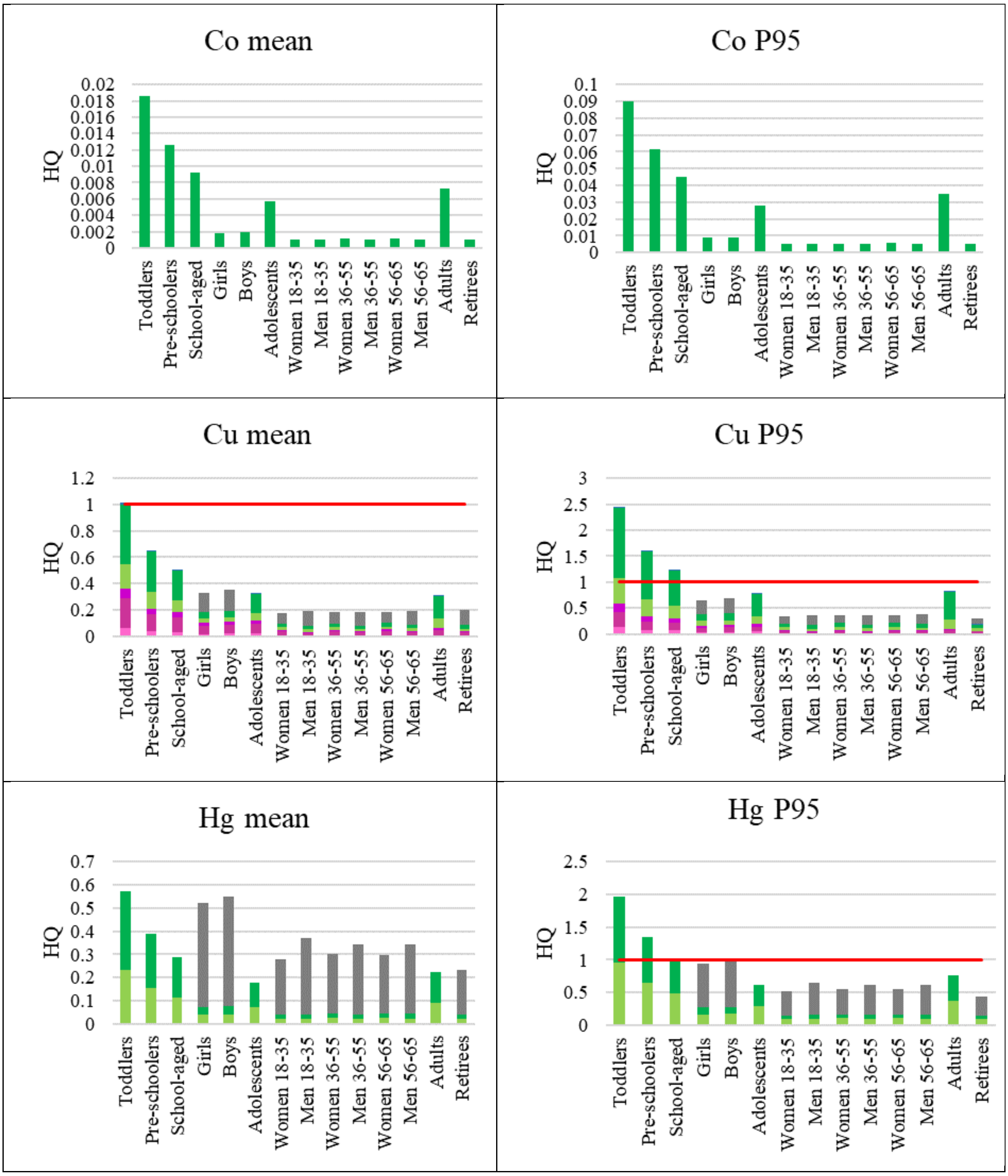
Co mean
HQ
0.02
0.018
0.016
0.014
0.012
0.01
0.008
0.006
0.004
0.002
0
Co P95
0.1
0.09
0.08
0.07
0.06
0.05
0.04
0.03
0.02
0.01
Cu mean
1.2
1
0.8
0.6
0.4
0.2
Cu P95
3
2.5
2
1.5
0.5
Hg mean
0.7
0.6
0.5
0.4
0.3
0.2
0.1
Hg P95
Toddlers
Pre-schoolers
School-aged
Girls
Boys
Adolescents
Women 18-35
Men 18-35
Women 36-55
Men 36-55
Women 56-65
Men 56-65
Adults
Retirees

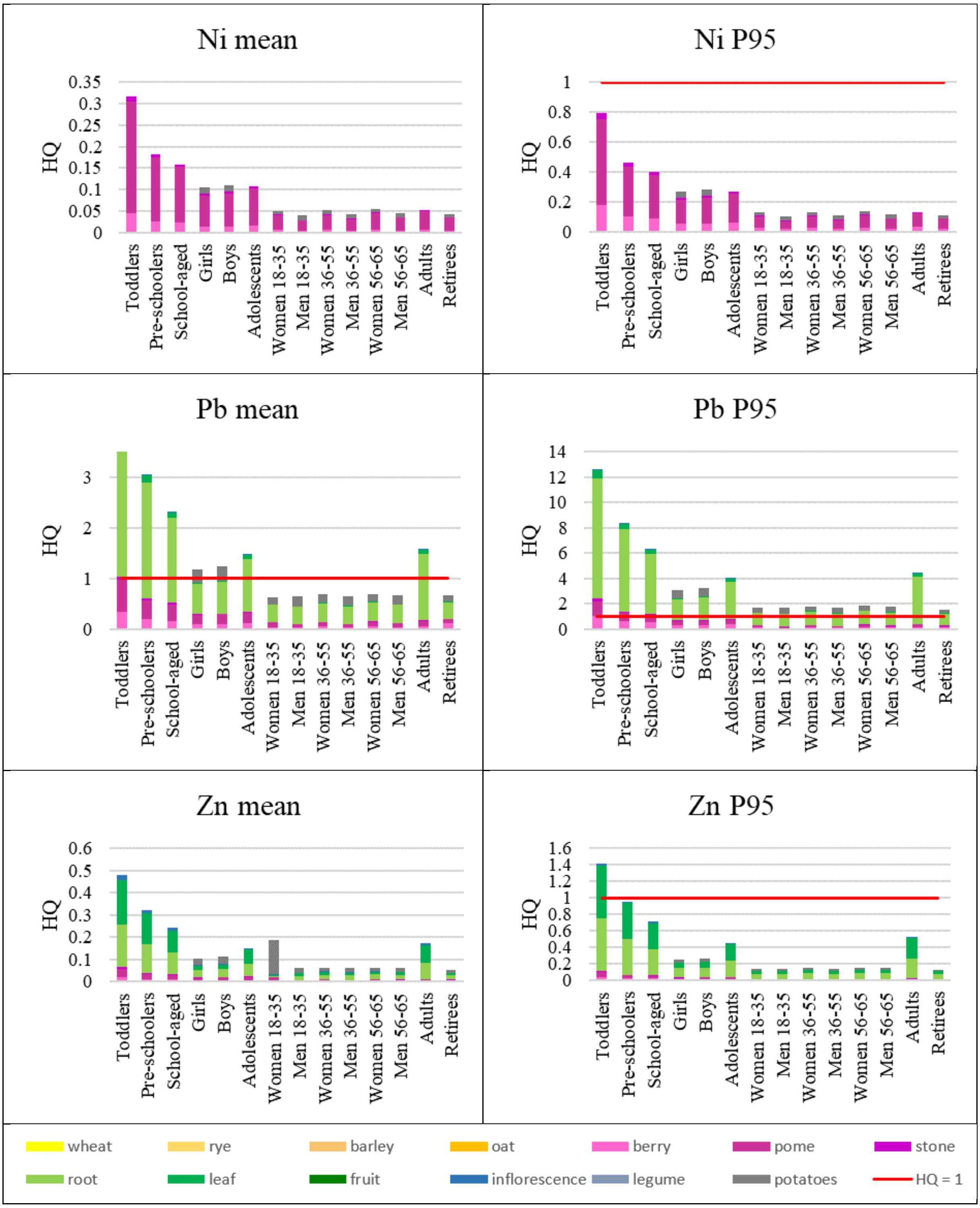

Supplementary Figure S1. Non-carcinogenic risk (HQ) values for consumed edible plants in individual voivodeships in Poland based on mean and P95 concentrations of PHEs; P95 – 95th percentile

References

1. Orzeł, D., Styczyńska, M., Biernat, J. Ocena zanieczyszczenia metalami ciężkimi produktów roślinnych z terenów uprzemysłowionych Dolnego Śląska. *Bromat. Chem. Toksykol.* **43(2),** 152-157 (2010).
2. Piekut, A., Baranowska R, Marchwińska-Wyrwał E, Ćwieląg-Drabek M, Hajok I, Dziubanek G, Grochowska-Niedworok E.. Is the soil quality monitoring an effective tool in consumers' protection of agricultural crops from cadmium soil contamination? - a case of the Silesia region (Poland). *Environ. Monit. Assess.* **190(25)**; 10.1007/s10661-017-6413-5 (2017).
3. Gut, K., Rogala, D., Marchwińska-Wyrwał, E. Exposure to cadmium among consumers of root vegetables cultivated in contaminated soils in Upper Silesia, Poland. *Med. Og. Nauk Zdr.* **23(4),** 245-249; 10.26444/monz/80448 (2017).
4. Bednarek, W., Tkaczyk, P., Dresler, S. Content of heavy metals as a criterion for assessing cucumber quality. (in Polish) *Acta Agrophys.* **10(2),** 273-285 (2007).
5. Majkowska-Gadomska, J., Dobrowolski, A., Mikulewicz, E., Francke, A. Concentrations of heavy metals and nitrates in eggplant grown with a biostimulator. *Pol. J. Environ. Stud.* **25(4),** 1787-1790; 10.15244/pjoes/62093 (2016).
6. Mania, M., Rebeniak, M., Postupolski, J. Exposure assessment of the population in Poland to the toxic effects of nickel from vegetable and their products. *Rocz. Panstw. Zakl. Hig.* **70(4),** 401-406; 10.32394/rpzh.2019.0095. (2019).
7. Czech, A., Pawlik, M., Rusinek, E. Contents of heavy metals, nitrates and nitrites in cabbage. *Pol. J. Environ. Stud.* **21(2),** 321-329 (2012).
8. Bielińska, E.J., Mocek-Płóciniak, A. Impact of geochemical soil conditions on selected heavy metals content in garden allotment Vegetables. *Pol. J. Environ. Stud.* **19(5),** 895-900 (2010).
9. Śmiechowska, M., Florek, A. Zawartość metali ciężkich w wybranych warzywach z uprawy konwencjonalnej, ekologicznej i działkowej. *J. Agric. Eng. Res.* **56(4),** 152-156 (2011).
10. Stępniowska, A., Czech, A., Sujak, A., Matusevicius, P., Chałabis-Mazurek, A. The effect of pumpkin varieties on the content of selected toxic elements from south-eastern Poland. *J. Food Compost. Anal.* **94,** 103632; 10.1016/j.jfca.2020.103632. (2020).
11. Grochowska-Niedworok, E., Nieć, J., Baranowska, R. Assessment of cadmium and lead content in tomatoes and tomato products. *Rocz. Panstw. Zakl. Hig.* **71(3),** 313-319; 10.32394/rpzh.2020.0126. (2020).
12. Gruszecka-Kosowska, A. Potentially Harmful Element Concentrations in the Vegetables Cultivated on Arable Soils, with Human Health-Risk Implications. *Int. J. Environ. Res. Public Health.* **16(20),** 4053; 10.3390/ijerph16204053. (2019).
13. Gruszecka-Kosowska, A. Human Health Risk Assessment and Potentially Harmful Element Contents in the Fruits Cultivated in the Southern Poland. *Int. J. Environ. Res. Public Health.* **16(24),** 5096; 10.3390/ijerph16245096. (2019).
14. Gruszecka-Kosowska, A. Human Health Risk Assessment and Potentially Harmful Element Contents in the Cereals Cultivated on Agricultural Soils. *Int. J. Environ. Res. Public Health.* **17(5),** 1674; 10.3390/ijerph17051674. (2020).

15. Borowiec, M., Huculak, M., Hoffmann, K., Hoffmann, J. Assessment of selected heavy metals content in plant food products in accordance with Polish law in force (in Polish). *PECO.* **3(2),** 433-438 (2009).
16. Grembecka, M., Szefer, P. Comparative assessment of essential and heavy metals in Fruits from different geographical origins. *Environ. Monit. Assess.* **185(11),** 9139-9160; 10.1007/s10661-013-3242-z (2013).
17. Kot, A., Zaręba, S., Wyszogrodzka-Koma, L. Assessment of lead contamination in cereals, cereal products and Potatoes from Lublin Region (in Polish). *Zywn-Nauk Technol. Ja.* **16(4),** 86-91 (2009).
18. Kot, A., Zaręba, S., Wyszogrodzka-Koma, L. Assessment of copper and zinc contents in selected cereal products (in Polish). *Bromat. Chem. Toksykol.* **44(1),** 32-37 (2011).
19. Wieczorek, J., Wieczorek, Z., Bieniaszewski, T. Cadmium and lead content in cereal grains and soil from cropland adjacent to roadways. *Pol. J. Environ. Stud.* **14(4),** 535-540 (2005).
20. Dymkowska-Malesa, M. Concentration of lead and cadmium in selected vegetables grown in the region of Warmia and Mazury. *Polish J. Food Nutr. Sci.* **4(57),** 119–121 (2007).
21. Figurska-Ciura, D., Łoźna, K., Styczyńska, M. Cadmium, lead, zinc and copper contents in selected vegetables and fruit from garden allotments of the south-western Poland. *Polish J. Food Nutr. Sci.* 2007, **4(57),** 137–143 (2007).
22. Dziubanek, G., Piekut, A., Rusin, M., Baranowska, R., Hajok, I. Contamination of food crops grown on soils with elevated heavy metals content. *Ecotoxicol. Environ. Saf.* **118,** 183–189; 10.1016/j.ecoenv.2015.04.032. (2015).
23. Dziubanek, G. *et al.* Cadmium in edible plants from Silesia, Poland, and its implications for health risk in populations. *Ecotoxicol. Environ. Saf.***,** 8–13; 10.1016/j.ecoenv.2017.03.048. (2017).
24. Bielińska, E. Influence of the Root layer on the content of cadmium and lead in soils and vegetable plants in regions with diverse anthropogenic impact (in Polish). *J. Agric. Eng. Res.* **54(3),** 16-20 (2009).
25. Mikuła, W., Indeka, L. Heavy metals in allotment gardens close to an oil refinery in Płock. *Wat. Air & Soil Poll.* **1–4(96),** 61–71; 10.1023/A:1026402218990. (1997).
26. Kapusta-Duch, J., Leszczyńska, T., Florkiewicz, A., Filipiak-Florkiewicz, A. Comparison of lead and cadmium contents in cruciferous vegetables grown under diversified ecological conditions: Cracow Region of Poland. *Ecol. Food Nutr.* **50(2),** 137-154; 10.1080/03670244.2011.552372. (2011).
27. Ognik, K, Rusinek, E., Sembratowicz, I. Truchliński, J. Contents of heavy metals, nitrate (V), and nitrate (III) in Fruits of elderberry and black chokeberry depending on harvest site and vegetation period. *Rocz. Panstw. Zakl. Hig.* **57(3),** 235–241. (2006).
